# The CCR4-NOT Complex Maintains Stability and Transcription of rRNA Genes by Repressing Antisense Transcripts

**DOI:** 10.1128/MCB.00320-19

**Published:** 2019-12-11

**Authors:** Shun Hosoyamada, Mariko Sasaki, Takehiko Kobayashi

**Affiliations:** a Institute for Quantitative Biosciences (IQB), Tokyo, Japan; b Department of Biological Sciences, Graduate School of Science, University of Tokyo, Tokyo, Japan; c Collaborative Research Institute for Innovative Microbiology, University of Tokyo, Tokyo, Japan

**Keywords:** CCR4-NOT complex, RNA degradation, genome instability, noncoding RNA, ribosomal RNA gene

## Abstract

The rRNA genes (rDNA) in eukaryotes are organized into highly repetitive gene clusters. Each organism maintains a particular number of copies, suggesting that the rDNA is actively stabilized. We previously identified about 700 Saccharomyces cerevisiae genes that could contribute to rDNA maintenance. Here, we further analyzed these deletion mutants with unstable rDNA by measuring the amounts of extrachromosomal rDNA circles (ERCs) that are released as by-products of intrachromosomal recombination. We found that extremely high levels of ERCs were formed in the absence of Pop2 (Caf1), which is a subunit of the CCR4-NOT complex, important for the regulation of all stages of gene expression. In the *pop2* mutant, transcripts from the noncoding promoter E-pro in the rDNA accumulated, and the amounts of cohesin and condensin were reduced, which could promote recombination events. Moreover, we discovered that the amount of rRNA was decreased in the *pop2* mutant. Similar phenotypes were observed in the absence of subunits Ccr4 and Not4 that, like Pop2, convey enzymatic activity to the complex. These findings indicate that lack of any CCR4-NOT-associated enzymatic activity resulted in a severe unstable rDNA phenotype related to the accumulation of noncoding RNA from E-pro.

## Abstract (precis)

The rRNA genes (rDNA) in eukaryotes are organized into highly repetitive gene clusters. Each organism maintains a particular number of copies, suggesting that the rDNA is actively stabilized. We previously identified about 700 Saccharomyces cerevisiae genes that could contribute to rDNA maintenance. Here, we further analyzed these deletion mutants with unstable rDNA by measuring the amounts of extrachromosomal rDNA circles (ERCs) that are released as by-products of intrachromosomal recombination.

## INTRODUCTION

All organisms have ribosomes that translate mRNA to protein. During the course of evolution, cellular demand for ribosomes has grown due to the increase in cell size and the enhanced number of cellular functions. Ribosomes consist of ribosomal proteins and RNA that occupy, in a cell of budding yeast Saccharomyces cerevisiae, ∼70% of the total protein and ∼60% of the total RNA (1). To sustain the synthesis of large numbers of ribosomes, the yeast cell has ∼150 tandemly repeated copies of rRNA genes (rDNA) on chromosome XII (chr. XII).

Repetitive sequences form unstable regions in a genome, as spontaneous recombination between repeats can lead to loss or gain of these sequences. The rDNA is one of the most abundant repetitive regions in eukaryotic genomes in which the copy number can vary, as has been observed in several organisms (2, 3). In spite of this unstable nature, each organism maintains its rDNA copy number at a particular, stable level, thereby suggesting that there are mechanisms at play that keep the overall repeat structure intact (4).

**FIG 1:**
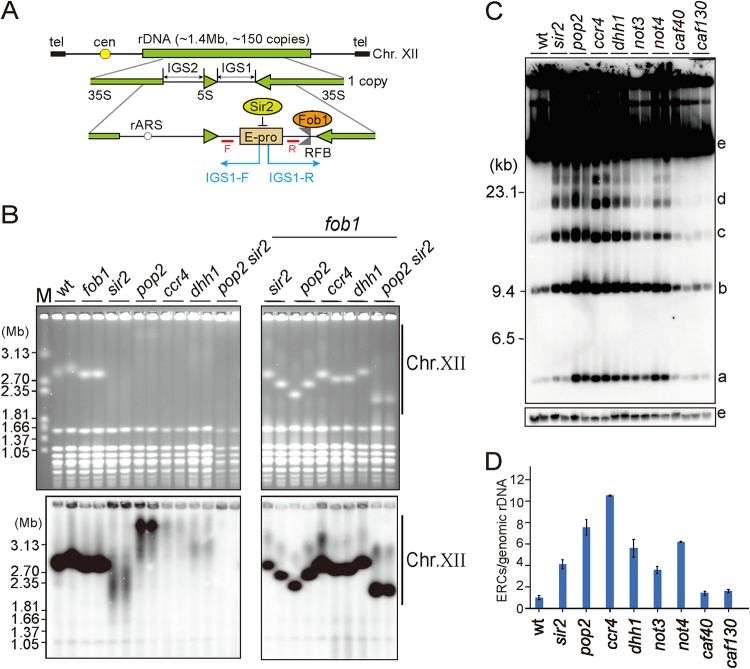
Mutants lacking components of the CCR4-NOT complex show rDNA instability. (A) The rDNA region in S. cerevisiae. The rDNA in S. cerevisiae is located on chromosome XII (chr. XII) as a tandem repeat. Tel, telomeres; cen, centromeres; IGS1 and IGS2, intergenic spacers; 35S, 35S rRNA; 5S, 5S rRNA; rARS (ribosomal autonomously replicating sequence), a replication origin; RFB, replication fork barrier. Arrows indicate the direction of transcription. IGS1-R and IGS1-F indicate the direction of noncoding transcription from the noncoding promoter, E-pro, and these transcripts were detected in experiments shown in Fig. 3 and 6 and Fig. S5 and S6. Red bars are the positions of Northern analysis probes. R and F are for IGS1-R and IGS1-F, respectively. (B) PFGE analysis in the single and double mutants of the CCR4-NOT complex with *fob1*. (Upper) Ethidium bromide (EtBr) staining. (Lower) Hybridization with an rDNA probe. M is the size marker (Hansenula wingei chromosomal DNA). (C) ERC assay in the CCR4-NOT complex mutants. ERCs were detected by Southern analysis as shown in Fig. S2A to K, but the genetic background is different. a, supercoiled monomer ERC; b, relaxed monomer ERC; c, supercoiled dimer ERC; d, relaxed dimer ERC; e, genomic rDNA. (D) Quantitation of ERCs in panel C. The signal intensities were measured and normalized by that of genomic rDNA as shown in Fig. S2. The values are relative to that of the wild-type strain. Error bars show the range from two independent experiments.

Some of the mechanisms that help to maintain the rDNA copy number are well studied in budding yeast (3). A major mechanism is gene amplification (5) (see Fig. S1 in the supplemental material). When rDNA copies are lost by intrachromosomal recombination, gene amplification occurs to compensate for the lost copies. The main players in the process of gene amplification are the replication fork barrier (RFB) site and the Fob1 protein (6, 7) (Fig. 1A). Fob1 bound at the RFB site inhibits replication fork progression in a polar fashion (8, 9), in the sense that it prevents head-on collision of the fork with the RNA polymerase I (PolI) transcription machinery. Some arrested forks are broken in the single-stranded regions (10), and the broken forks become one-ended double-strand breaks (DSBs) that can trigger repair by homologous recombination (Fig. S1). During gene amplification, the broken end is repaired by unequal sister chromatid recombination. This process is regulated by histone deacetylase Sir2 and a noncoding bidirectional promoter, E-pro, which is present in the intergenic region between the genes for 5S and 35S (11, 12) (Fig. 1). In cells with low rDNA copy numbers, E-pro is activated and noncoding transcripts are generated, which prevents association of cohesin with the rDNA (11) and facilitates gene amplification (Fig. S1). When the copy number reaches ∼150, Sir2 represses E-pro and cohesin can associate with rDNA, which only permits repair by equal sister chromatid recombination. This type of recombination does not change the copy number. The precise regulation of amplification and mechanisms that maintain the repeat stability, however, are not well elucidated yet.

To reach a better understanding of how rDNA is maintained in yeast, we previously examined the rDNA stability in ∼4,800 gene deletion mutants and classified them by rDNA stability into four ranks, rank 1 (more stable than the wild type [wt]), rank 2 (as stable as the wt), rank 3 (more unstable than the wt), and rank 4 (extremely unstable) (13). Around 700 mutants with unstable rDNA (of ranks 3 and 4) were identified, and some of these have been analyzed in more detail (13–15). The possibility that among these ~700 mutants, identified in a first step to select genes important for rDNA stability from ∼4,800 candidates, there will be false positives cannot be excluded (16). Therefore, any follow-up analysis requires confirmation of the rDNA instability in these mutants, which we have done for about one-third of the mutants categorized in rank 3. We reexamined 242 of these strains by pulsed-field gel electrophoresis (PFGE), and 73 strains were subjected to more quantitative assays that assessed the level of extrachromosomal rDNA circles (ERCs) that are produced by recombination in the rDNA and, thus, are indicative of rDNA instability (17, 18) (Fig. S1). Among the mutants examined, a mutant lacking one of the components of the CCR4-NOT complex (19–23), Pop2 (Caf1), produced an extremely high level of ERCs. The rDNA was also unstable in mutants lacking other members of the complex (*ccr4*, *dhh1*, *not3*, and *not4*). The CCR4-NOT complex is a multifunctional protein complex that is involved in the regulation of gene expression of various classes of RNA: mRNA, rRNA, and other non-protein-coding RNA (24–27). The deadenylase activity of Pop2 and Ccr4 can trigger mRNA degradation by exonucleases such as Rrp44 (28).

Here, we report that in the *pop2* and *ccr4* mutants, levels of noncoding RNA transcribed from E-pro are highly increased, and the extent of association to rDNA by cohesin and condensin was reduced. Moreover, the amount of rRNA in the *pop2* mutant is reduced to about half of the wild-type level. These mutant phenotypes depended on the presence of the E-pro promoter. We conclude that the CCR4-NOT complex mediates the degradation of noncoding RNA transcribed from the E-pro promoter and in this manner contributes to maintaining rDNA stability and rRNA synthesis.

## RESULTS

### Screening of mutants with unstable rDNA.

To confirm rDNA instability and identify mutants with highly unstable rDNA, we conducted a secondary screen with 242 mutants out of ∼660 categorized as rank 3 in our previous screen that lack genes whose products function in DNA replication, recombination, repair, and transcription according to the gene ontology annotation in the *Saccharomyces* Genome Database (29, 30). First, genomic DNA prepared from these mutants was separated by PFGE, and rDNA stability was analyzed by comparing the degree of smearing of the chr. XII band in a mutant to that of wild-type chr. XII. Of the 242 strains examined, for 73 strains the rDNA instability phenotype was reproduced and deletion of the genes of interest was confirmed (data not shown). These strains were then subjected to more quantitative ERC assays. Genomic DNA, prepared from mutant strains that were grown from two independent single colonies, was separated by conventional agarose gel electrophoresis, and ERCs were detected by Southern blotting (see Fig. S2 in the supplemental material). As controls, we included DNA isolated from the wild-type strain and also from the *sir2* mutant that is known to produce a high level of ERCs (31). The amount of ERCs was normalized to the amount of genomic rDNA to correct for differences in rDNA copy number. More ERCs accumulated in 62 mutants than in wild-type cells (Fig. S2L). Among them, the *pop2* mutant contained the highest level of ERCs, which was ∼50-fold higher than that of the wild type and ∼4-fold higher than that of the *sir2* mutant (Fig. S2). Pop2 is a component of the CCR4-NOT complex (20, 21). Interestingly, two other members of this complex, Ccr4 and Dhh1, had originally also been classified as rank 3 (13). Unfortunately, both genes were omitted from the ERC assay, as *DHH1* did not pass the selection by gene ontology and a deletion of *CCR4* was not in the library used for the secondary screen. Overall, these findings indicate that the CCR4-NOT complex plays an important role in maintaining rDNA stability.

### The CCR4-NOT complex is required for the maintenance of rDNA stability.

To examine the contribution of the CCR4-NOT complex to the maintenance of rDNA stability, we constructed mutants lacking components of this complex in the W303 genetic background, which is different from the BY4741 background used for the yeast knockout library, and analyzed rDNA stability in these mutants by PFGE. Of the ten subunits in the CCR4-NOT complex (27), we could not establish deletion mutants for *NOT1*, *NOT2* or *NOT5*, suggesting that these genes are essential in the W303 background. The mutants lacking Pop2, Ccr4, Dhh1, Not3, or Not4, but not Caf40 or Caf130, showed smeared bands of chr. XII, as seen in the *sir2* mutant, after staining with ethidium bromide (EtBr), indicating that the rDNA copy number frequently changes in these strains and that their rDNA is unstable (Fig. 1B; see also Fig. S3 in the supplemental material). Consistent with these results, these mutants produced a high level of ERCs (Fig. 1C and D). In addition, compared to the wild-type strain or the *sir2* mutant, the size of chr. XII, and therefore the rDNA copy number, were increased in all these strains except the *not3* mutant (Fig. 1B and Fig. S3).

To examine the relationship between rDNA recombination and the CCR4-NOT complex, we established double mutants with *fob1*. In the absence of Fob1, replication fork arrest and DSB formation that can induce recombination do not occur and rDNA becomes stable (10, 12, 15). The chr. XII band in each of the double mutants was as sharp as that in the *fob1* single mutant (Fig. 1B and Fig. S3), suggesting that the CCR4-NOT complex functions downstream of Fob1. In other words, the CCR4-NOT complex maintains rDNA stability after replication fork arrest and DSB formation.

Pop2 and Ccr4 are generally considered to have deadenylase activities that can promote cytoplasmic mRNA degradation (32, 33). However, whether Pop2 has a catalytic function is still unclear. In yeast, the catalytic activity of Pop2 is not required for mRNA poly(A) removal (34), while Not4 is a ubiquitin protein ligase (35, 36). Dhh1 interacts with the CCR4-NOT complex and has helicase activity (37, 38). Thus, the absence of CCR4-NOT-associated enzymatic activity, by deleting Dhh1 or one of the subunits Ccr4, Pop2, and Not4, results in a severely unstable rDNA phenotype, namely, a large but variable increase in rDNA copy number and extensive ERC formation.

### Deadenylase activity of CCR4-NOT is required to maintain rDNA stability.

**FIG 2:**
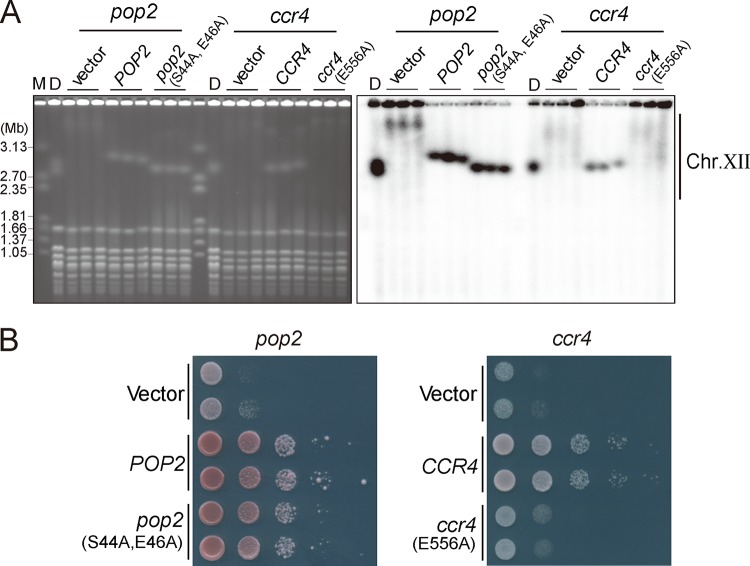
Effect of deadenylase activity on rDNA stability and growth. rDNA stability (A) and growth (B) were tested in the *pop2* and *ccr4* deletion mutants transformed by empty vector or plasmid carrying the indicated genes by PFGE (A) and by spot assay (B). *pop2* (S44A, E46A) and *ccr4* (E556A) carry mutations that abolish the deadenylase activity. M is a size marker (*H. wingei* chromosomal DNA). D indicates diploid strains that were transformed with each plasmid from which the analyzed haploid strains were derived.

Defects in deadenylation activity in the *pop2* and *ccr4* mutants may cause the observed rDNA instability. To test this possibility, we examined the effect of point mutations in *POP2* or *CCR4* that would abolish enzymatic activity on rDNA stability (39–42). We introduced an empty plasmid, a plasmid carrying wild-type *POP2* or *CCR4*, or plasmids carrying catalytically dead alleles of *pop2* (S44A, E46A) or *ccr4* (E556A) into the diploid strain heterozygous for *pop2* or *ccr4* gene deletion, followed by tetrad dissection to isolate the haploid clones of interest. As seen before (Fig. 1B), in the *pop2* mutant carrying the empty plasmid, large, smeared chr. XII bands were observed (Fig. 2A). In contrast, expression of wild-type or catalytically dead Pop2 proteins in *pop2* mutant cells did not exhibit the rDNA instability seen in the *pop2* mutant (Fig. 2A), suggesting that the presence, rather than the catalytic activity, of Pop2 is important for maintaining rDNA stability. Expression of wild-type Ccr4 in the *ccr4* mutant suppressed the rDNA instability (Fig. 2A). The rDNA in the *ccr4* mutant expressing the catalytically inactive Ccr4-E556A protein, however, was unstable and similar to the rDNA in the mutant carrying an empty plasmid. Thus, Ccr4 activity is essential for rDNA maintenance, while Pop2 has a mediating role. Indeed, the presence of Pop2, not its activity, is found to be required for tethering Ccr4 to the complex (21, 43, 44). Thus, in the *pop2* deletion mutant, the complex loses both Pop2 and Ccr4, and thereby all deadenylase activity, leading to rDNA instability.

After complementation of the deletion strains (*pop2* and *ccr4*) by reintroducing wild-type or catalytically inactive alleles, growth is restored in the case of Pop2 but only barely so in the case of Ccr4 (Fig. 2B). This result confirms the rDNA analysis and points to a correlation of a functional CCR4-NOT complex with the recovery of rDNA stability (Fig. 2A). Interestingly, rDNA instability by itself does not affect growth (see below).

### Noncoding transcripts accumulate in CCR4-NOT mutants.

According to the current model for the mechanism of rDNA recombination (Fig. S1), rDNA stability is mainly affected by three events: (i) replication fork arrest, (ii) DSB formation, and (iii) noncoding transcription from E-pro. To identify which event is linked to a malfunctioning CCR4-NOT complex, we first determined the levels of arrested forks at the RFB site in the *pop2*, *ccr4*, and *dhh1* mutants, in which the unstable rDNA phenotype was most prominent. To this end, replication intermediates were analyzed by two-dimensional (2D) gel electrophoresis (6, 45) in which arrested replication forks at the RFB site appear as a strong signal along the arc of Y-shaped replication intermediates. The levels of arrested forks in the CCR4-NOT mutants were similar to that in the wild type (Fig. S4A to C). DSBs at the RFB site were detected by one-dimensional gel electrophoresis (15), which provided an indication for a partial correlation between the DSB levels and rDNA instability (Fig. S4D and E), that is, levels of DSBs were ∼1.6-fold higher in the *pop2* and *ccr4* mutants than in the wild type, but no difference was observed for the *dhh1*, *not3*, and *not4* mutants. Although these increases in DSB levels may be a cause of rDNA instability in these mutants, the difference seems too small to explain, for example, the large increase of ERCs in the *pop2* mutant.

**FIG 3:**
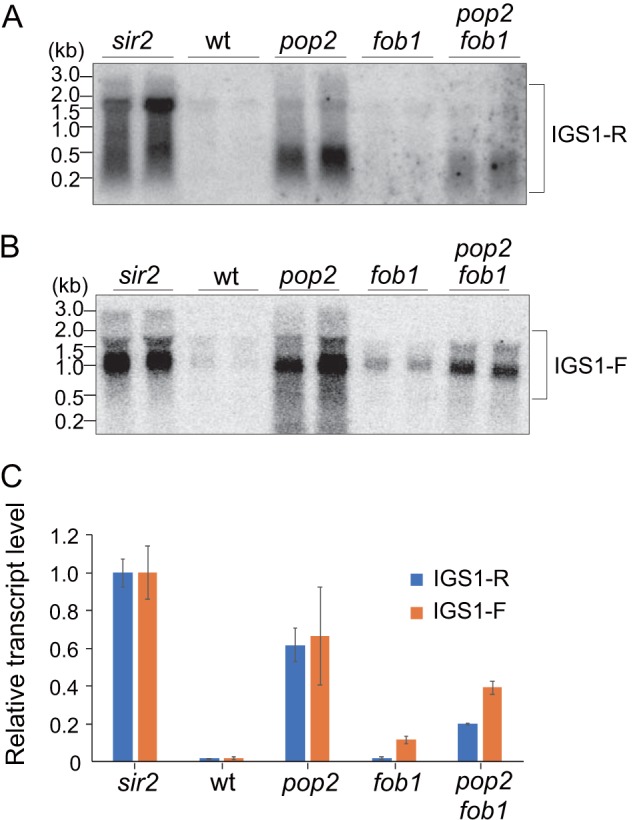
*pop2* mutant accumulates noncoding transcripts from E-pro. (A and B) Northern analysis of E-pro transcripts. E-pro transcripts, IGS1-R (A) and IGS1-F (B), were detected in samples prepared from two independent cultures for each genotype, using probes for IGS1-R (A) and IGS1-F (B) (Fig. 1A). (C) Quantification of the levels of IGS1-R (A) and IGS1-F (B). The signal intensity of transcripts was normalized to the total amount of RNA. Relative transcript levels normalized to that in the *sir2* mutant (set to 1) are shown. Error bars show deviations from the means for the duplicates.

The noncoding transcripts from E-pro were detected by Northern blot analysis (Fig. 3A and B). Transcripts in either direction accumulated in the *sir2* and *pop2* mutants relative to the levels in wild-type cells, in which they were barely detectable. Quantitation of the amount of E-pro-derived transcripts showed that these markedly increased in the *pop2* mutant compared to those in the wild type (Fig. 3C). A strong accumulation of transcripts initiated at E-pro was also observed in the absence of other subunits, in particular the enzymatic subunits of the CCR4-NOT complex (Fig. S5A to F). In addition, in the *pop2 sir2* double mutant more E-pro-derived transcripts were formed than produced in each single mutant (Fig. S6), which was also observed for the *sir2* double mutant with *dhh1* (Fig. S5C and D). These results indicate that on top of reduced E-pro suppression in the absence of Sir2, the synthesized transcripts that are derived from E-pro are not degraded in the CCR4-NOT mutants when the enzymatic activity of the complex is compromised or are more efficiently produced. The accumulation of these noncoding transcripts is linked to a dramatically reduced rDNA stability.

### E-pro transcripts increase rDNA instability in CCR4-NOT complex mutants.

**FIG 4:**
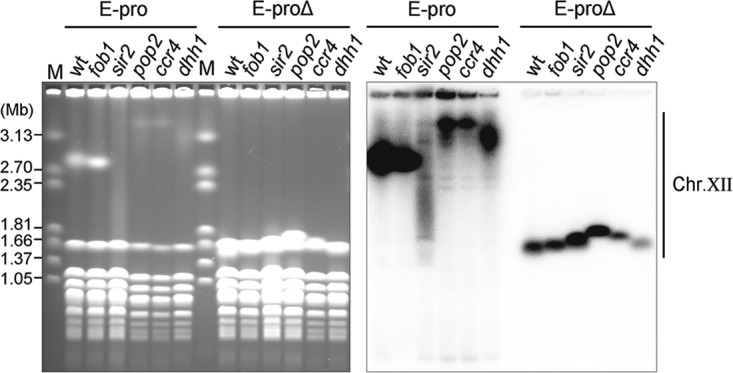
rDNA instability in the CCR4-NOT complex mutants is dependent on E-pro. PFGE analysis of the CCR4-NOT complex mutants with or without E-pro. In the E-proΔ strain, E-pro was replaced with the bidirectional *GAL1/10* promoter. These strains were grown in the presence of raffinose, which does not activate the *GAL1/10* promoter (E-proΔ). (Left) The gel was stained by EtBr. (Right) Hybridization with an rDNA-specific probe. M is a size maker (*H. wingei* chromosomal DNA).

To find out whether reduced rDNA stability in the *pop2* mutant is directly related to the noncoding transcription from E-pro itself, we established the *pop2* deletion in a mutant lacking the E-pro sequence from its rDNA repeats (11). In this strain, E-pro was replaced with the *GAL1/10* promoter that is not induced in medium containing raffinose as the carbon source and not regulated by Sir2. In raffinose-containing medium, the rDNA in this strain is stable with fewer ERCs, but in galactose-containing medium the opposite results are observed (46). The absence of Pop2 in this E-pro-less (E-proΔ) strain, when grown in raffinose-containing medium, did not affect rDNA stability, as shown by PFGE (Fig. 4). The band of chr. XII in the *E-proΔ pop2* mutant was as sharp as that in wild-type and *fob1* cells. The presence of Ccr4 or Dhh1, other CCR4-NOT complex subunits required for maintaining stable rDNA, also is not essential for rDNA maintenance in the absence of E-pro (Fig. 4). In contrast, in galactose-containing medium, where transcription of the E-pro locus is activated in E-proΔ cells, the absence of CCR4-NOT complex subunits Pop2 and Ccr4 caused smearing and lengthening of chr. XII bands (Fig. S7). In the *dhh1* mutant, however, the chr. XII band is more comparable to that in wild-type and *sir2* cells. These results indicate that transcripts from E-pro that accumulate in the absence of the deadenylase subunits of the CCR4-NOT complex increase rDNA instability.

### Increased E-pro transcription in the absence of Pop2 leads to reduced rDNA association of cohesin and condensin.

**FIG 5:**
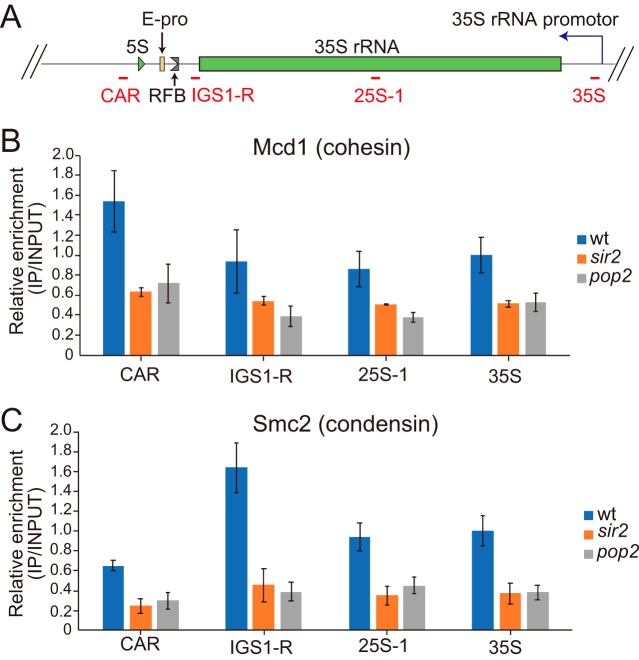
Reduced rDNA association of cohesin and condensin in the *pop2* mutant. (A) Schematic overview of the positions of qPCR amplicons (red). (B and C) The levels of cohesin (Mcd1) (B) and condensin (Smc2) (C) associated with the rDNA were determined by ChIP-qPCR analyses in strains carrying FLAG-tagged *MCD1* (B) and *SMC2* (C), respectively. Relative fold enrichment was indicated by calculating the [rDNA (IP)/CUP1 (IP)]/[rDNA (WCE)/CUP1 (WCE)] ratio (56). The values were relative to those for wt 35S pre-rRNA. The error bars indicate standard errors of the means from three independent experiments.

Transcription from E-pro, which is increased in the *sir2* mutant, prevents cohesin from associating with rDNA, resulting in decreased rDNA stability (12). We tested whether a similar scenario occurs in the *pop2* mutant by chromatin immunoprecipitation (ChIP) (47). In this assay, protein associated with DNA is precipitated and the DNA is detected by real-time PCR. To analyze rDNA association of cohesin (Mcd1), we used a FLAG-tagged version of the protein and four PCR primer sets that amplify various regions in the rDNA (Fig. 5A). In the *pop2* mutant, the rDNA association of Mcd1 is comparable to that in the *sir2* mutant and reduced compared to that of the wild type (Fig. 5B). We also tested condensin (Smc2), the dissociation of which from rDNA is linked to rDNA instability (48). Using FLAG-tagged Smc2, the association with rDNA was, compared to that of the wild-type strain, reduced by more than 2-fold in the *pop2* and *sir2* mutants (Fig. 5C). These results indicate that increased levels of E-pro transcription inhibit cohesin and condensin association with rDNA and reduce rDNA stability.

### Accumulation of E-pro-derived transcripts interferes with rRNA synthesis.

**FIG 6:**
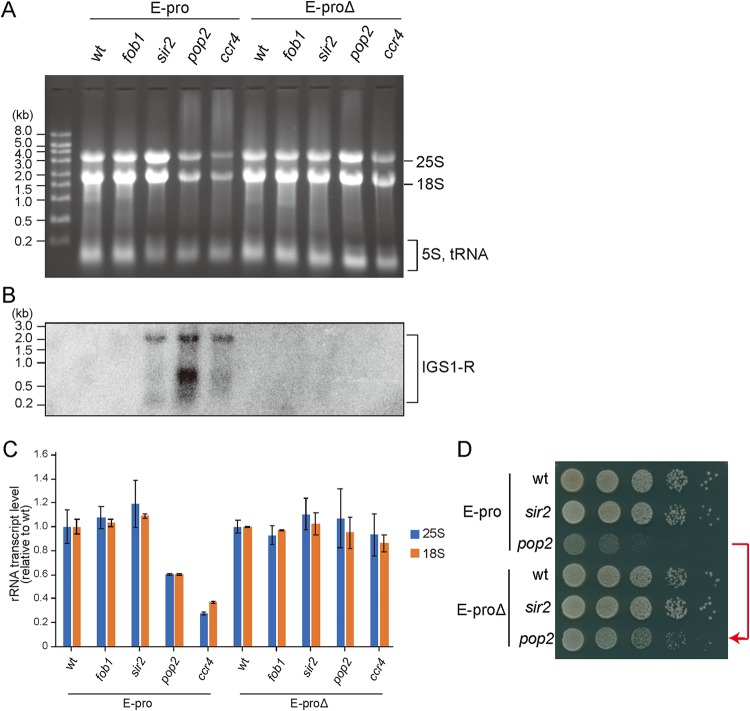
Levels of rRNAs are reduced in the *pop2* and *ccr4* mutants in an E-pro-dependent manner. (A) rRNA transcripts were analyzed by electrophoresis in the indicated strains carrying E-pro sequence (E-pro) or *GAL1/10* promoter in place of E-pro (E-proΔ). rRNA was detected by EtBr staining. 18S, 25S, and 5S rRNA transcripts were indicated. The position of 5S rRNA includes tRNA and other small RNAs. (B) Northern analysis to detect noncoding transcripts in the indicated strains. With the IGS1-R probe (Fig. 1A), transcripts from E-pro were detected. As the *GAL* promoter is not activated in raffinose-containing medium, the transcript was undetectable in the E-proΔ strain. The same gel as that used in panel A was hybridized. (C) The quantitation of rRNA transcripts in panel A. Each signal intensity was normalized to total RNA. The values are relative to that of the wt. Error bars show the range from two independent experiments. (D) Growth of the *pop2* and *sir2* mutants was determined by a spot assay on raffinose-containing medium.

Enhanced E-pro transcription in CCR4-NOT mutants could interfere with the synthesis and processing of the 35S rRNA precursor. To see whether the RNA polymerase I and II machineries collide, we measured rDNA transcripts. As shown in Fig. 6A to C, the amount of 25S and 18S rRNA in the *pop2* and *ccr4* mutants was reduced about 2-fold compared to the wild-type level or that of the *sir2* mutant. Moreover, the amount of preprocessed 35S rRNA was also reduced (Fig. S8B), suggesting that the absence of enzymatic active CCR4-NOT interfered with rDNA transcription and/or processing. To confirm whether this reduction of rRNA transcripts is related to the activity of E-pro, we measured the levels of rDNA transcripts in the *E-proΔ pop2* mutant that was grown in noninducing medium. This replacement resulted in more wild-type-like amounts of 18S and 25S rRNA (Fig. 6A and C), while accumulation of IGS1-R transcripts was no longer detectable (Fig. 6B). Moreover, the poor growth in the *pop2* mutant partially recovered in the *E-proΔ pop2* mutant (Fig. 6D; also see below).

Apart from colliding transcription machines, it is possible that the transcripts from E-pro form R-loops (DNA/RNA hybrids) that inhibit 35S transcription. To test this possibility, we precipitated DNA/RNA hybrids with S9.6 antibody from wild-type and *pop2* mutant cells and checked by quantitative PCR (qPCR) for hybrid formation at various sites on the rDNA. As shown in Fig. S9, the amount of DNA/RNA hybrids detected in the *pop2* mutant was 2-fold lower than that for the wild type at any site. Therefore, R-loop formation seems not to be related to reduced rDNA transcription and, while often associated with genome instability, not instrumental in the rDNA instability phenotypes of the *pop2* mutant.

### Accumulation of E-pro-derived transcripts is related to growth in CCR4-NOT complex mutants.

In the *pop2* mutant, E-pro transcripts increase and rDNA becomes unstable, like that in the *sir2* mutant (Fig. 1B and 3). The major difference between the *pop2* and *sir2* mutants is that the *pop2* mutant grows poorly compared to the wild-type strain and the *sir2* mutant (Fig. 6D). The effect on cell growth by accumulation of noncoding RNA transcribed from E-pro could be compared to a situation where less noncoding RNA was formed from an Sir2-independent, noninduced *GAL* promoter that replaced the E-pro (E-proΔ) (Fig. 6D). This experiment revealed that the growth defect observed in the absence of Pop2 was alleviated in the *E-proΔ* strain, suggesting that accumulation of E-pro transcripts is related to a cause of the observed growth defect.

Concomitant with an accumulation of E-pro transcripts, the *pop2* mutant has about 3-fold more rDNA (Fig. S5G) and 8- to ∼50-fold more ERCs (Fig. 1D and Fig. S2L) than the wild type. Although not observed for the *sir2* mutant, such an increase in unstable rDNA may be a burden and could affect cell growth. Therefore, we tested the growth of the *pop2 fob1* double mutant, which has rDNA that is stably kept at the normal rDNA copy level (Fig. 1B). In the double mutant, ERCs did not accumulate (Fig. S10A and B), but despite this decrease in rDNA stress, cell growth in the absence of Pop2 and Fob1 was still poor (Fig. S10C). In contrast to the absence of Sir2, CCR4-NOT mutations could affect a variety of processes that support removal of unwanted compounds (possibly E-pro-derived transcripts). Therefore, our results suggest that increases in rDNA copy and ERCs are not a major cause of poor growth of CCR4-NOT mutants. Most likely, the accumulation of products that normally are cleared by processes controlled by this complex reduces the production of rRNA and ribosomes, causing poor growth.

## DISCUSSION

**FIG 7:**
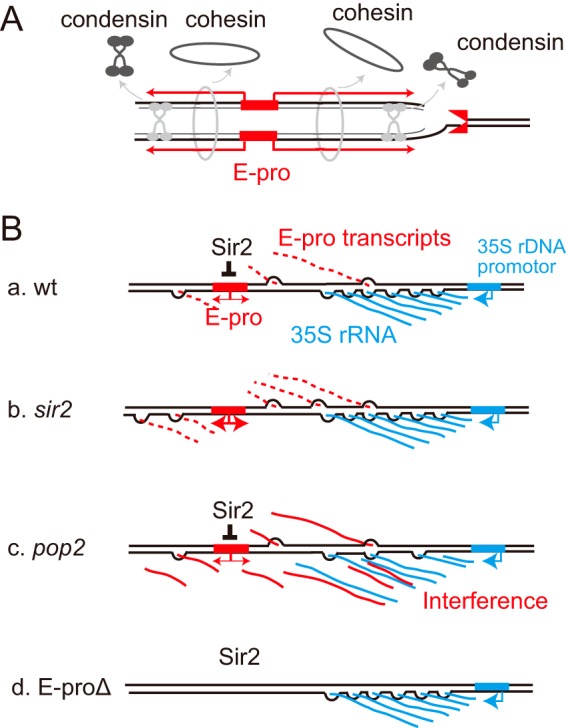
Models of how E-pro transcription affects rDNA stability and transcription. (A) In the *sir2* or RNA degradation-defective mutants, E-pro transcription promotes dissociation of cohesin and condensin, facilitating unequal sister chromatid recombination and rDNA instability after DSB formation at the RFB site. (B) Model of rDNA transcription interference by noncoding RNA. (a) In the wild-type strain, Sir2 represses E-pro and the small amount of transcripts is degraded by the exosome and/or the CCR4-NOT complex. (b) In the *sir2* mutant, a large amount of transcripts is synthesized but degraded with little to no interference of 35S synthesis. (c) In the *pop2* mutant, undegraded E-pro transcripts accumulate and interfere with the rDNA transcription and/or maturation of the pre-rRNA into ribosomes. (d) In the absence of the E-pro-driven transcription (E-proΔ), no interference of rDNA transcription occurs.

rRNA is one of the most abundant RNAs in organisms. During the course of evolution, cells have developed systems to produce large amounts of rRNA transcripts. One of them is gene amplification of rDNA. In the budding yeast, the noncoding promoter E-pro regulates the type of recombination that supports rDNA amplification (11) (see Fig. S1 in the supplemental material). We found that in the *pop2* mutant rDNA becomes unstable and that the E-pro transcripts accumulate, which interferes with rDNA association of cohesin and condensin. As a result, unequal sister chromatid recombination is expected to increase and to induce rDNA instability (12, 48) (Fig. 7A). Moreover, an accumulation of E-pro transcripts was observed to lead to a reduction of rRNA production only in the absence of Pop2 or Ccr4, not Sir2 (Fig. 6). Whether this reduction would have caused a growth defect, as seen upon destabilizing RNA polymerase I (49), or other processes are affected by CCR4-NOT complex mutations remains to be seen. We did observe, however, an improvement in cell growth when E-pro transcription was reduced, indicating that accumulation of E-pro transcripts is part of the problem these mutant cells face.

The CCR4-NOT complex functions to mediate degradation of mRNA through its deadenylation activity (22, 50–52), which in essence is a 3′-5′-exoribonuclease activity. Here, we found that the complex may also be required for the degradation of noncoding RNAs that are transcribed from E-pro in the rDNA. It has been shown that transcripts from E-pro are degraded by the exosome and the TRAMP complex containing Trf4 (53). Trf4 is a poly(A) polymerase component of the TRAMP complex that mediates nuclear RNA degradation by the exosome. Additionally, it has been shown that the 3′ end of IGS1-R is deadenylated by Pap1 (53). The CCR4-NOT complex can interact with components of TRAMP and the nuclear exosome during the degradation of noncoding nuclear RNAs, such as misprocessed and polyadenylated snoRNAs (24). Moreover, it has been shown that CCR4-NOT subunits are physically enriched in the rDNA (26). Therefore, it is feasible that the CCR4-NOT complex also functions, together with TRAMP and the nuclear exosome, in the degradation of noncoding RNA transcribed from E-pro in the rDNA. However, in the *trf4* mutant, only the transcripts made in the direction of a replication fork that would be colliding with 35S transcription (IGS1-R) accumulated (53). In the CCR4-NOT mutants (*pop2*, *ccr4*, *dhh1*, and *not4*), we observed the accumulation of transcripts produced in both directions from the E-pro (Fig. 3 and Fig. S5). In fact, for recombination that leads to rDNA amplification, bidirectional transcription from the E-pro is required (11). This may be the reason that rDNA stability is not obviously reduced in the *trf4* mutant (53). Our analysis of the yeast deletion collection also showed that rDNA in the *trf4* mutant and in exosome mutants was as stable as that in the wild-type strain (13). Therefore, we speculate that one of the reasons the rDNA integrity is so severely affected in CCR4-NOT mutants is that the removal of E-pro transcripts is more seriously impaired in these strains than in the *trf4* or exosome mutants.

Accumulation of E-pro transcripts is also observed in the *sir2* mutant, in which rDNA is unstable (Fig. 3) (11). In contrast to the CCR4-NOT mutants (*pop2* and *ccr4*), in the case of the *sir2* mutant, cell growth and the amount of rDNA transcripts were normal (Fig. 6 and Fig. S8). In the *sir2* mutant, E-pro transcription is not repressed and more RNA is transcribed (11) (Fig. 3 and Fig. S5 and S6) but can be cleared out by CCR4-NOT-dependent processes. In the *pop2* mutant the transcripts accumulate, presumably because they are not degraded (Fig. 7B). E-pro transcripts may anneal with 35S rRNA and interfere with its transcription and/or promote its degradation. The exact length of E-pro transcripts that run against the direction of 35S transcription (IGS1-R) remains unclear, because the transcription does not appear to terminate at a specific site while a huge amount of 18S rRNA appears to comigrate and could interfere with Northern analyses (Fig. 6A and B). Still, the noncoding transcripts could be long enough to anneal to large sections of 35S rRNA. In the *pop2* mutant but not in the *sir2* mutant (where the E-pro transcripts can be degraded), the accumulated antisense RNA may interfere with rRNA production when such hybrids are formed and not sufficiently untangled (Fig. 7B, panel c). Further study is required to elucidate how the CCR4-NOT complex controls the degradation of E-pro transcripts.

The deadenylase-defective allele of *pop2* (S44A, E46A) (41) had a small effect on rDNA stability and growth compared to that of *ccr4* (E556A) (40) (Fig. 2). This suggests that the deadenylase activity of Pop2 is less important for CCR4-NOT function than that of Ccr4 (32). There is structural evidence that Pop2 has a role to connect Ccr4 to the complex (23), so that in the *pop2* deletion mutant not only Pop2 but also Ccr4 is absent from the complex. In the case of the deadenylase-defective Pop2 (S44A, E46A), however, it is possible that the protein can hold on to Ccr4 and keeps the main deadenylase activity linked to the complex. In the deletion- or deadenylase-defective mutants of Ccr4, only the enzymatic activity of Pop2 resides in the complex, which is not sufficient to maintain rDNA stability and normal growth (Fig. 2), as is supported by other reports (44, 54).

The rDNA transcriptional interference by antisense transcription from E-pro, which leads to reduced rRNA synthesis, could explain another phenotype caused by a defective CCR4-NOT complex in rDNA maintenance. In addition to unstable rDNA in the *pop2* and *ccr4* mutants, their rDNA copy number is significantly increased (Fig. 1B and Fig. S5G). This suggests that cells have a means to sense the amount of rRNA, which feeds back to stimulate rDNA amplification when rRNA production is down. Consistent with this hypothesis, it has been shown that the levels of nascent rRNA are increased in CCR4-NOT complex-defective mutants by PolI ChIP analysis (26). If more copy numbers produce more E-pro transcripts, then these transcripts could interfere with rDNA transcription to the extent that amplification recombination remains induced. This ongoing rDNA amplification would result in a huge amount of rDNA copies on the chromosome and, upon recombination, as ERCs. Further analysis is required to unravel these possibilities.

## MATERIALS AND METHODS

### Yeast strains, oligonucleotides, and growth conditions.

Mutant yeast strains used for ERC assays, shown in Fig. S2 in the supplemental material, are derived from the Yeast Knockout Collection (YSC1053; Open Biosystems). The wild-type strain shown in Fig. S2 is BY4741 (*MAT***a** *his3-1 leu2-0 met15-0 ura3-1*). Other yeast strains used in this study are derivatives of NOY408-1b, which is derived from W303, and are listed in Table S1. Gene deletion was performed by one-step gene replacement. Oligonucleotides used in this study are listed in Table S2.

For PFGE and ERC assays, cells were grown in YPD medium (1% yeast extract, 2% peptone, and 2% glucose) at 30°C overnight. For PFGE shown in Fig. 4 and Fig. S7, cells were grown in YPRaffinose (1% yeast extract, 2% peptone, and 2% raffinose) or YPGalactose (1% yeast extract, 2% peptone, and 2% galactose). To prepare RNA, cells were grown overnight at 30°C in YPAD medium (YPD containing 1.67% adenine). To perform 2D and DSB analyses, cells were first grown in 5 ml of YPD at 30°C overnight until saturation. Cells were then inoculated into 30 to 50 ml fresh YPD medium at a density of 0.5 × 10^6^ to 1.0 × 10^6^ cells/ml and grown until the culture reached a density of 2 × 10^6^ to 3 × 10^6^ cells/ml. To perform ChIP analysis, cells were first grown in 5 ml of YPAD at 30°C overnight until saturation. Cells were then inoculated into 50 ml of fresh YPAD at an optical density at 600 nm (OD_600_) of 0.1. Cells were incubated until the culture reached an OD_600_ of 0.8 to 1.0 (log phase).

### Plasmid construction.

YCplac33-*POP2* and YCplac33-*CCR4* were constructed by the In-Fusion cloning method as follows. The genomic regions containing either the *POP2* or the CCR4 open reading frame plus ∼500 bp upstream and downstream were PCR amplified from S. cerevisiae genomic DNA using primers HS405 and HS406 (in the case of *POP2*) or HS409 and HS410 (for *CCR4*). Linearized YCplac33 vector fragments were generated by PCR amplification of YCplac33 using primers HS411 and HS412 (*POP2* specific) or HS413 and HS414 (*CCR4* specific). The gene and vector fragments with 18 bases of homology at each end were fused using the In-Fusion HD cloning kit (TaKaRa) according to the manufacturer’s instructions. The reaction mixes were used for transformation of Escherichia coli DH5α, and plasmid DNA from positive clones was analyzed by Sanger sequencing.

Plasmids YCplac33-*pop2* (S44A, E46A) and YCplac33-*ccr4* (E556A) were constructed using the PrimeSTAR mutagenesis basal kit (TaKaRa) according to the manufacturer’s instructions. Briefly, primers were designed to have 15 nucleotides of overlap at their 5′ ends and to contain the mutations that change Pop2 serine 44 into alanine and glutamate 46 into alanine (primers HS533 and HS534) or that changes Ccr4 glutamate 556 into alanine (primers HS535 and HS536). These primers were used to perform PCR using YCplac33-*POP2* or YCplac33-*CCR4* as a template. The resulting PCR products were transformed into E. coli DH5α. Plasmid DNA was sequenced to confirm that the mutations had been introduced.

### Genomic DNA preparation.

For PFGE, 2D gel analysis, DSB analysis, and ERC assays, genomic DNA was prepared in low-melting-temperature agarose plugs as described previously (15). For 2D gel analysis and DSB analysis, cells were collected and treated with 0.1% sodium azide prior to genomic DNA preparation. To assess rDNA copy numbers, genomic DNA was prepared by the conventional method as described previously (15).

### ERC assays.

One-half of an agarose plug was cut and electrophoresed on 0.4% agarose gel in 1× Tris-acetate-EDTA (40 mM Tris base, 20 mM acetic acid, and 1 mM EDTA, pH 8.0) at 1.0 V/cm for 48 h at 4°C with buffer circulation. The buffer was changed after ∼24 h of electrophoresis. DNA was transferred to Hybond-N^＋^(GE Healthcare), followed by Southern blot hybridization as described previously (15). The probe to detect genomic rDNA and ERCs was PCR amplified from S. cerevisiae genomic DNA using primers HS204 and HS205. Quantification of signals was performed by Fujifilm MultiGauge, version 2.0, software (Fujifilm). The membranes were first exposed to phosphor screens so that signals did not become saturated. Scanned images were used to quantify genomic rDNA. The membranes were then exposed for several days, and scanned images were used to quantify supercoiled monomers, relaxed monomers, supercoiled dimers, and relaxed dimers. The sum of ERC signals was normalized to signals of genomic rDNA. For each panel (blot) in Fig. S2A to K, the mean value for the wt was set to 1. For Fig. S2L, ERC levels in all the mutants examined were combined. As a representative ERC score for *sir2*, the value shown in Fig. S2B was used.

### PFGE.

One-third of an agarose plug and Hansenula wingei chromosomal DNA markers (Bio-Rad) were electrophoresed on 1.0% agarose gel (pulsed-field certified agarose; Bio-Rad) in 0.5× Tris-borate-EDTA (TBE) buffer (44.5 mM Tris base, 44.5 mM boric acid, and 1 mM EDTA, pH 8.0) on a Bio-Rad contour-clamped homogeneous electric field DR-III system under the following conditions: 68 h at 3.0 V/cm at 14°C, 120° included angle, initial switch time of 300 s, and final switch time of 900 s. DNA was transferred to Hybond XL, followed by Southern blot hybridization as described previously (15). The probe to detect chr. XII was PCR amplified from S. cerevisiae genomic DNA using primers HS210 and HS211.

### 2D gel electrophoresis.

To detect replication intermediates, 2D neutral/neutral gel electrophoresis was performed as described previously (45). Briefly, one-third of an agarose plug was equilibrated four times in 1 ml Tris-EDTA (TE; pH 9.0) for 15 min and then equilibrated twice with 1 ml of 1× H buffer (TaKaRa) for 20 min and digested in 200 μl of 1× H buffer containing 150 U of BglII (TaKaRa) for 8 to 16 h at 37°C. DNA was separated on 0.4% agarose gel (Star Agarose; Rikaken) in 1× TBE at 1.32 V/cm for 14 h at room temperature. DNA was stained with 0.3 μg/ml of EtBr. Gel slices containing replication intermediates in rDNA were excised, rotated 90°, and separated on 1.2% agarose gel in 1× TBE containing 0.3 μg/ml of EtBr at 6.0 V/cm for 6 h at 4°C. DNA was transferred to Hybond-N^+^ (GE Healthcare), followed by Southern blot hybridization as described previously (15). The probe to detect replication intermediates was prepared by PCR amplification of S. cerevisiae genomic DNA using primers HS266 and HS275. Quantification was performed with ImageJ (NIH). We determined the signal for the RFB spot (*a*) and the intensity of the replication intermediate with the spots for 1N and 2N (*b*). The relative replication-intermediate levels were calculated as *a* divided by *b*. The score was normalized to the wt value of 1.

### DSB analysis.

To assess DSB frequency, physical assays were performed as described previously (15). Briefly, one-third of an agarose plug was equilibrated four times in 1 ml TE for 15 min, equilibrated twice with 1 ml of 1× NEBuffer 3.1 for 30 min, and digested in 150 μl of 1× NEBuffer 3.1 containing 150 U of BglII (New England Biolabs) for 12 to 16 h at 37°C. DNA was separated on 0.7% agarose gel in 1× TBE at 2.0 V/cm for 20 to 22 h at room temperature with buffer circulation. DNA was transferred to Hybond XL (GE Healthcare), followed by Southern blot hybridization as described previously (15). The probe used for DSB analysis was PCR amplified from S. cerevisiae genomic DNA using primers HS206 and HS207. Quantification was performed by Fujifilm MultiGauge, version 2.0, software. Signals of DSB fragments and replication intermediates containing arrested forks were quantified. The level of DSBs was assessed by normalizing DSB signals to signals of replication intermediates containing arrested forks. The values for the wt were set to 1.

### rDNA copy number assays.

Approximately 5 μg of genomic DNA was digested with 50 U of BglII (TaKaRa). DNA was separated on 1% agarose gel at 10 V/cm for 30 to 40 min. DNA was transferred to Hybond N^+^ (GE Healthcare), followed by Southern blot hybridization as described previously (15). Quantification was performed by ImageJ (NIH). The membranes were first hybridized by probes to detect *MCM2*, which was PCR amplified using primers HS542 and HS543. Probes were stripped and the membranes were hybridized with an rDNA probe that was PCR amplified using primers HS393 and HS394. Signals of the rDNA fragment were normalized to that of the *MCM2* fragment. The mean values for the wt were set to 1.

### Yeast RNA preparation.

Cells were collected and washed with ice-cold water treated with 0.1% diethylpyrocarbonate. Cells were resuspended in 400 μl of TES (10 mM Tris-HCl [pH 7.5], 10 mM EDTA at pH 7.5, 0.5% SDS). An equal volume of acidic phenol, a mixture consisting of equal volumes of phenol and 50 mM sodium acetate at pH 4.0, was added to the cell suspension, followed by vigorous vortexing for about 10 s. Cells were incubated at 65°C for 1 h, incubated for 5 min on ice, and centrifuged at 17,400 × *g* for 10 min at 4°C. The aqueous phase was reextracted with 200 μl of acidic phenol and 200 μl of chloroform, after which the RNA was isolated by ethanol precipitation. RNA was quantified by measuring the OD_260_ using a NanoDrop ND-1000 spectrophotometer (Thermo Fisher Scientific).

### Northern blotting and hybridization.

Either 10 μg of total RNA (to detect noncoding RNA from IGS1) or 1 to 5 μg of total RNA (to detect rRNA) and, as a size standard, DynaMarker RNA High (BioDynamics Laboratory Inc.) were separated on 1% agarose gel containing 6% formaldehyde and 1× morpholinepropanesulfonic acid (MOPS; 20 mM MOPS, 2 mM sodium acetate, 1 mM EDTA, 0.1% diethylpyrocarbonate, adjusted to pH 7.0 with NaOH) in 1× MOPS running buffer at 10 V/cm for 30 to 40 min. RNA was transferred to Hybond N^+^ (GE Healthcare).

To prepare strand-specific probes used for Northern blot hybridization, 10 ng of PCR product was mixed with the primer at a final concentration of 0.1 μM in a reaction volume of 17.25 μl. The PCR product was heat denatured by boiling for 3 min at 95°C, followed by gradual cooling to 50°C, and then 2.5 μl of 10× *Ex Taq* buffer, 0.25 μl of *Ex Taq* (1.25 U; TaKaRa), 2.5 μl of 0.2 mM deoxynucleoside triphosphate mixture, and 2.5 μl of [α-^32^P]dCTP (3,000 Ci/mmol; Perkin Elmer) was added and the mixture was incubated at 72°C for 15 to 20 min. Unincorporated labeled nucleotides were removed using ProbeQuant G-50 microcolumns (GE Healthcare). Noncoding RNA from IGS1 was detected by hybridization overnight at 65°C using Rapid-hyb buffer (GE Healthcare), and rRNA was detected by hybridization overnight at 65°C using ULTRAhyb ultrasensitive hybridization buffer (Thermo Fisher Scientific). Quantification was performed by Fujifilm MultiGauge, version 2.0, software to quantify results of Northern blotting and ImageJ (NIH) to quantify rRNA levels in gels stained with EtBr. Each signal intensity of rRNA was normalized to that of total RNA. The transcript levels are relative to those of the wt (set to 1). Primers to obtain PCR products used as a template for linear PCR yielding the labeled probes were the following: for the 25S rRNA probe, HS391 and HS392; for 18S rRNA, HS387 and HS388; for 35S rRNA, HS397 and HS398; for IGS1-F transcripts, HS274 and HS275; and for IGS1-R transcripts, HS266 and HS296. Primers used for probe synthesis by linear PCR are the following: for the 25S rRNA probe, HS392; for 18S rRNA, HS388; for 35S rRNA, HS398; to detect IGS1-F transcripts, HS275; and to detect IGS1-R transcripts, HS266.

### ChIP.

ChIP was performed as described previously, with some modifications (47). Cells at an OD_600_ equivalent to 50 to 60 were collected and cross-linked with 1% formaldehyde at room temperature for 15 min, and formaldehyde was quenched for 5 min after adding glycine to a final concentration of 125 mM. Cells were washed twice with cold PBS (137 mM NaCl, 2.7 mM KCl, 10 mM Na_2_HPO_4_, 1.8 mM KH_2_PO_4_, pH 7.4) and resuspended in 600 μl lysis buffer (50 mM HEPES-KOH at pH 7.5, 500 mM NaCl, 1 mM EDTA at pH 8.0, 1% Triton X-100, 0.1% sodium deoxycholate, 0.1% SDS, and cOmplete, EDTA free) with 0.5-mm-diameter zirconia beads (YZB05; Yasui Kikai) in 2-ml screwcap tubes. Cells were disrupted by vigorous shaking for 20 cycles of 30 s at 2,700 rpm followed by 30 s at rest at 4°C in a Multibeads shocker (Yasui Kikai). Disrupted cells were collected and centrifuged at 700 × *g* for 10 min at 4°C. The pellet with the chromatin fraction was resuspended in 300 μl of lysis buffer. Chromatin was sheared by sonication at 4°C for 10 cycles of 30 s on and 30 s off on the high setting on a Bioruptor (Cosmo Bio). Samples were centrifuged at 3,800 × *g* for 2 min at 4°C. Twenty microliters of lysate was set aside as input DNA.

Ten microliters of Dynabeads protein G (no. 10004D; Thermo Fisher) was prewashed with PBS containing 5 mg/ml bovine serum albumin (BSA). Beads were incubated with 2.0 μg of antibody in 200 μl of PBS containing 5 mg/ml BSA at 4°C for 2 h, followed by washing the beads with lysis buffer. RNA-DNA hybrids were precipitated using S9.6 antibody (MABE1095; Merck). Mcd1-Flag and Smc2-Flag were precipitated using anti-FLAG antibody (F3165; Sigma). Beads were incubated with a mixture of 120 μl of lysate and 120 μl of lysis buffer at 4°C overnight. Beads were washed twice with lysis buffer at 4°C for 5 min, once with wash buffer (10 mM Tris-HCl [pH 8.0], 250 mM LiCl, 0.5% Nonidet P40 [IGEPAL-CA630; Sigma], 0.5% Na-deoxycholate, 1 mM EDTA [pH 8.0], and cOmplete mini EDTA-free proteinase inhibitor cocktail [Sigma]) at 4°C for 10 min and once with 1× TE (pH 8.0) at 4°C for 1 min. Immunoprecipitates were eluted by incubating beads with 120 μl of TE containing 1% SDS at 65°C for 10 min. The supernatants were saved. Beads were washed with 130 μl of TE containing 1% SDS, and the supernatants were combined with the first eluates. Cross-links were reversed from input and IP samples by incubating samples at 65°C overnight. Samples were incubated with 10 μl of 20 mg/ml proteinase K at 50°C for 2 h. Finally, DNA was isolated by phenol-chloroform extraction and ethanol precipitation. Isolated DNA was resuspended in 100 μl of TE.

### qPCR and quantification methods.

Quantitative PCR (qPCR) was set up using SYBR Premix *Ex Taq* (TaKaRa) in a total volume of 10 μl containing 0.5 μl of DNA (1:3 dilution of IP samples and 1:30 dilution of input) and primers at a final concentration of 0.3 μM in 1× master mix. qPCR was performed on a thermal cycler dice real-time system II (TaKaRa) under the following conditions: denaturation at 95°C for 30 s, followed by 40 cycles of 95°C for 5 s and 60°C for 30 s. Primers used for qPCR are the same as those used in a previous study (55): 35S, HS431 and HS432; 18S, HS435 and HS436; 25S-1, HS445 and HS446; 25S-2, HS449 and HS450; IGS1-R, HS453 and HS454; 5S, HS457 and HS458; CAR, HS459 and HS460; and *CUP1*, CUP1-fw and CUP1-rv. Relative fold enrichment was indicated by calculating the [rDNA (IP)/CUP1 (IP)]/[rDNA (WCE)/CUP1 (WCE)] ratio (56). The value of the wt was normalized to 1.

### Spot assay.

For spot assays, cells were grown in SC-ura (synthetic complete medium minus uracil) (Fig. 2B) or YPRaffinose (Fig. 6D) at 30°C overnight. Cells were diluted to 10^7^ cells/ml (first well) and then further diluted 10-fold (2nd well), 10^2^-fold (3rd well), 10^3^-fold (4th well), and 10^4^-fold (5th well). Ten microliters of each dilution was spotted on a plate, which was incubated at 30°C for 2 days.

## Supplementary Material

Supplemental file 1
