# Supplementary material for: The CCR4-NOT Complex Maintains Stability and Transcription of rRNA Genes by Repressing Antisense Transcripts
Source: Mol Cell Biol. 2019 Dec 11;40(1):e00320-19. doi: 10.1128/MCB.00320-19 (PMC6908257; doi:10.1128/MCB.00320-19)
Supplement: Supplemental file 1 [file MCB.00320-19-s0001.pdf]

SUPPLEMENTARY DATA

SUPPLEMENTARY FIGURE LEGENDS

Figure S1. rDNA recombination model

Three copies of rDNA during replication (S1). See text for details.

Figure S2. ERC assay for detection of rDNA unstable mutants.

(**A-K**) (Upper panels) ERCs were detected by Southern analysis. Positions of supercoiled monomer (**a**), relaxed monomer (**b**), supercoiled dimer (**c**) and relaxed dimer (**d**) ERCs, and genomic rDNA (**e**) are indicated. Two independent colonies were tested. M is the size marker (λ HindIII digest). (Lower panels) Quantitation of upper panel. The signal intensities were measured and normalized to that of genomic rDNA. The values are relative to that of wt. Error bars show range of two independent experiments. (**L**) Relative amount of ERC to wt. The ERC values from **A-K** were ranked in an ascending order, with the wt value indicated by a red arrow.

Figure S3 Stability of rDNA in CCR4-NOT complex mutants.

PFGE analysis in the single and double mutants of the CCR4-NOT complex with *fob1*. (Upper panel) Ethidium bromide (EtBr) staining. (Lower panel) hybridization to an rDNA probe. M is the size marker (*Hansenula wingei* chromosomal DNA).

Figure S4. RFB and DSB activity in the CCR4-NOT complex mutants.

(**A**)Two dimensional (2D) gel electrophoresis for RFB activity. The red arrows indicate the position of RFB spots. (**B**) Schematic explanation of 2D gel electrophoresis results. (**C**) Quantitation of RFB activity. The signal intensities of RFB spots were measured and normalized to those of the replication intermediates. The error bars are S.E.M.

from three independent experiments. (**D**) One dimensional gel electrophoresis for DSB activity. The position of DSB bands, linear fragments and arrested forks are indicated. (Lower panel) more exposed image showing a better contrast for the phosphorimager signals around the DSB bands. (**E**) Quantitation of DSB activity. The signal intensities of DSB bands were measured and normalized to those of arrested fork signals. The error bars are S.E.M. from three independent experiments. *p*-values of *t*-test are 0.024(*pop2*), 0.046 (*ccr4*), 0.954 (*dhh1*), 0.202 (*not3*), 0.227 (*not4*).

Figure S5. E-pro transcription in the CCR4-NOT complex mutants and their rDNA copy numbers.
(**A**)(**C**)(**E**) Northern analysis for E-pro transcripts. E-pro transcripts were detected as in Figure. 2. (**B**) Quantitation of (**A**). (**D**) Quantitation of (**C**). (**F**) Quantitation of (**E**). Each signal intensity was normalized to total RNA. The values are relative to that of the *sir2* mutant. Error bars show a range of two independent experiments. (**G**) Copy number of rDNA in each mutant was measured by determining the amount of rDNA relative to a single-copy gene *MCM2* by Southern analysis. The values for copy number variation are relative to the wild-type copy number. Error bars show range of two independent experiments.

Figure S6. E-pro transcription in *sir2 pop2* double mutants
(**A**) E-pro transcripts were detected by Northern analysis. IGS1-R (upper panel) and IGS1-F (lower panel) transcripts are shown. (**B**) Quantitation of (**A**). Each signal intensity was measured and normalized to total RNA. The values are relative to that of the *sir2* mutant. Error bars show a range of two independent experiments.

Figure S7. rDNA instability in the CCR4-NOT complex mutants is dependent on E-pro. PFGE analysis of the CCR4-NOT complex mutants with the bi-directional *GAL1/10*

promoter instead of the original E-pro (E-proΔ). These strains were grown in the presence of raffinose or galactose, conditions where transcription is either not expressed or induced, respectively. (Left panel) the gel was stained by EtBr. (Right panel) hybridization to an rDNA-specific probe. M is a size maker (*H. wingei* chromosomal DNA).

Figure S8. Detection of rRNA in the *pop2*, *fob1* and *sir2* mutants.
(**A**) Ribosomal RNA (rRNA) was detected in the EtBr stained gel as in Figure 5A. (**B**) The 35S rRNA was detected by Northern analysis with an ITS specific probe that recognizes the unprocessed 35S rRNA. The gel used in (**A**) was transferred for hybridization. (**C**) Quantitation of (**A**)(**B**). rRNAs (35S, 25S, and 18S RNA) were quantitated and each signal intensity was normalized to the total RNA. The values are relative to the wild type strain. Error bars show the range of two independent experiments. (**D**) rRNA was detected by hybridization with specific probes for 25S, 18S and 5S. (**E**) Quantitation of (**D**). The signal intensities were quantitated and normalized to the total RNA. The values are relative to wt. Error bars show the range of two independent experiments.

Figure S9. Detection of RNA/DNA hybrids.
(**A**) Overview of positions of qPCR amplicons. (**B**) RNA/DNA hybrids were precipitated with anti RNA/DNA antibodies and detected by qPCR. Relative fold enrichment was indicated by calculating the ratio of [rDNA (IP)/CUP1 (IP)]/[rDNA (WCE)/CUP1 (WCE)] (S4). The values were relative to those for the wild type. The error bars indicate S.E.M of three independent experiments.

Figure S10. ERC assay and growth in *fob1*, *pop2* and their double mutant.
(**A**) ERC assay for *fob1*, *pop2* and their double mutant. ERCs were detected by Southern analysis as in Figure 1C and Supplementary Figure S2A-K. (**B**) Quantitation

of ERCs in (**A**). The signal intensities were measured and normalized to the genomic rDNA as in Supplementary Figure S2. The values are relative to that of wild-type strain. The error bars indicate S.E.M of three independent experiments. (**C**) Growth of *fob1*, *pop2* and their double mutant was determined by a spot assay.

SUPPLEMENTARY TABLES

Table S1. *S. cerevisiae* strains used in this study

Table S2. Oligonucleotides used in this study

SUPPLEMENTARY REFFERENCES

S1. Kobayashi, T. (2006) Strategy to maintain the stability of the ribosomal RNA gene repeats. *Genes Genet Systems*, **81**, 155-161.

S2. Nogi, Y., Yano, R., and Nomura, M. (1991) Synthesis of large rRNAs by RNA polymerase II in mutants of *Saccharomyces cerevisiae* defective in RNA polymerase I. *Proc Natl Acad Sci U S A*, **88**, 3962-3966.

S3. Kobayashi, T. and Ganley, A.R. (2005) Recombination regulation by transcription-induced cohesin dissociation in rDNA repeats. *Science (New York, N.Y.)*, **309**, 1581-1584.

S4. Huang, J., and Moazed, D. (2003) Association of the RENT complex with nontranscribed and coding regions of rDNA and a regional requirement for the replication fork block protein Fob1 in rDNA silencing. *Genes Dev,***17**, 2162-2176.

Hosoyamada_FigS1

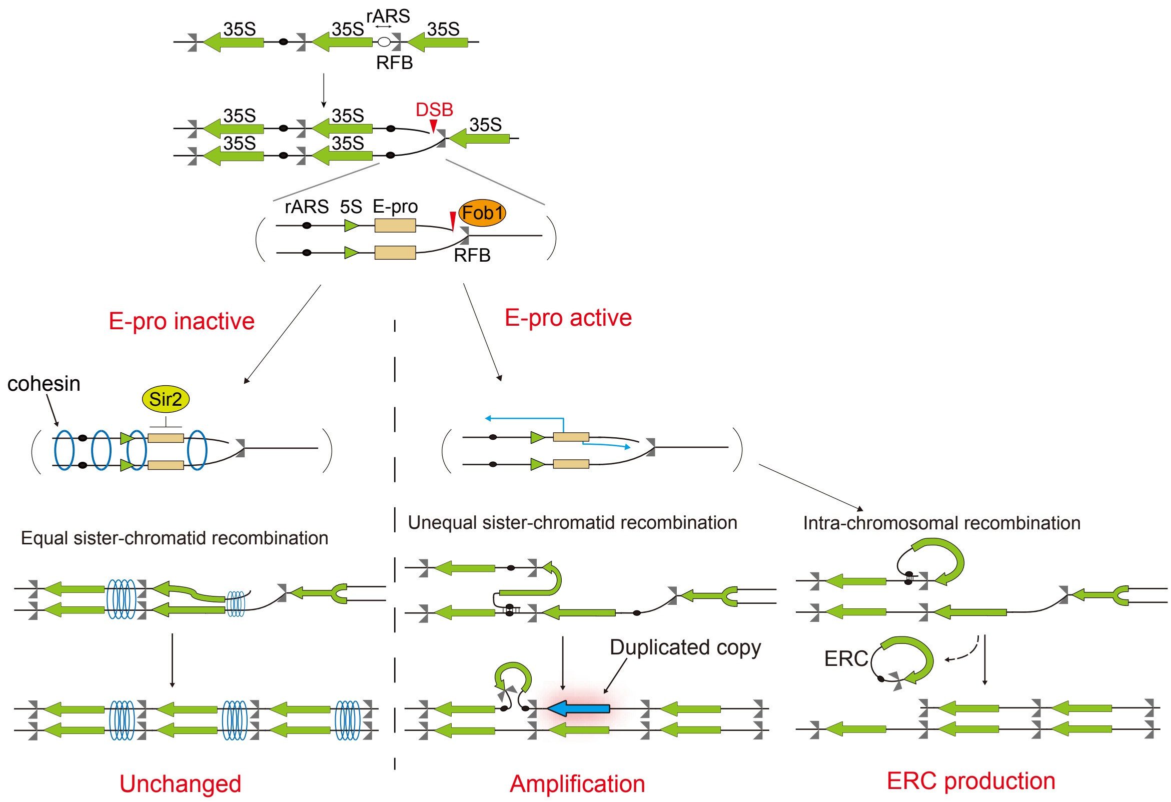

Hosoyamada_FigS2A, B, C, D, E, F

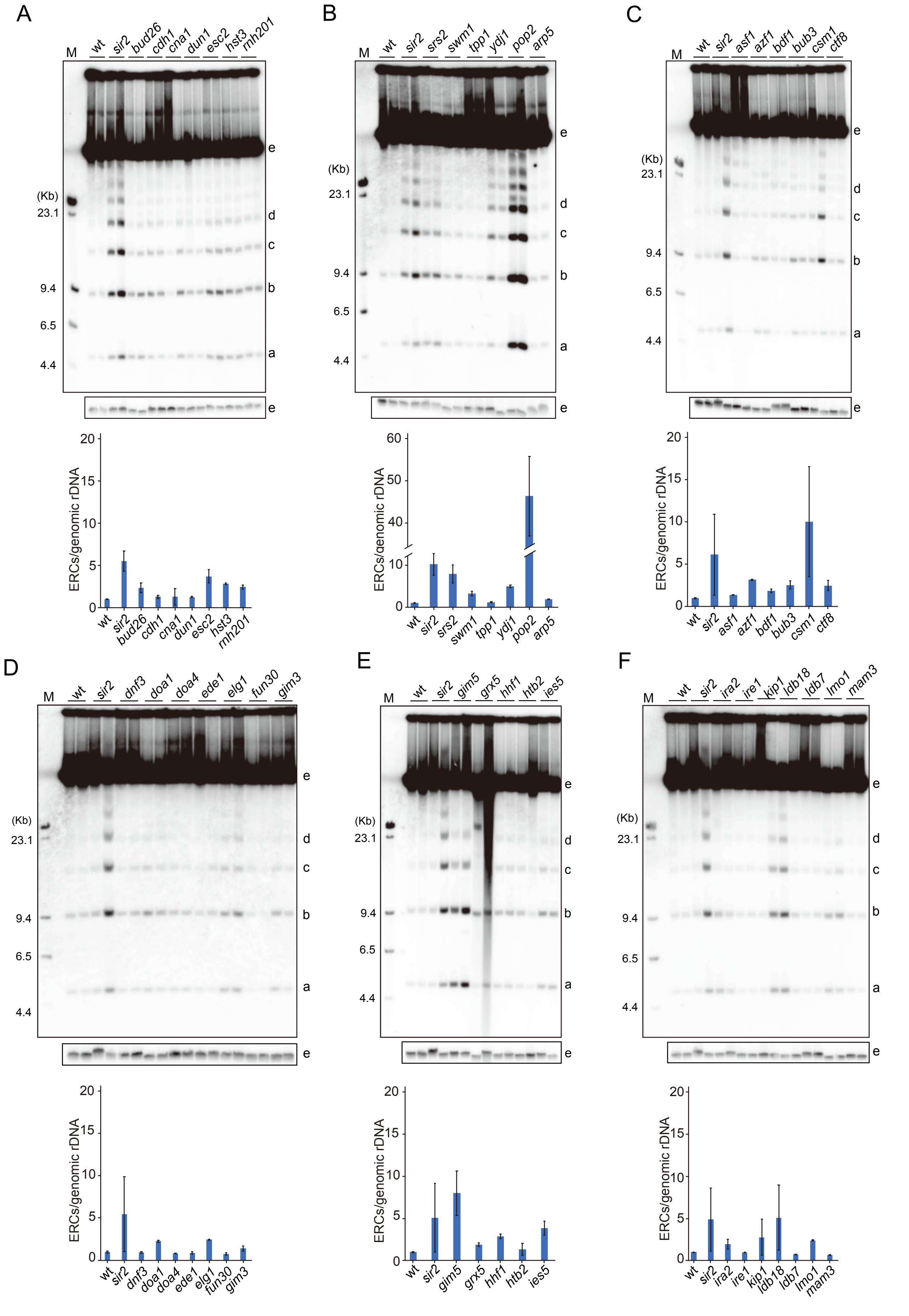

Hosoyamada_FigS2G, H, I, J, K

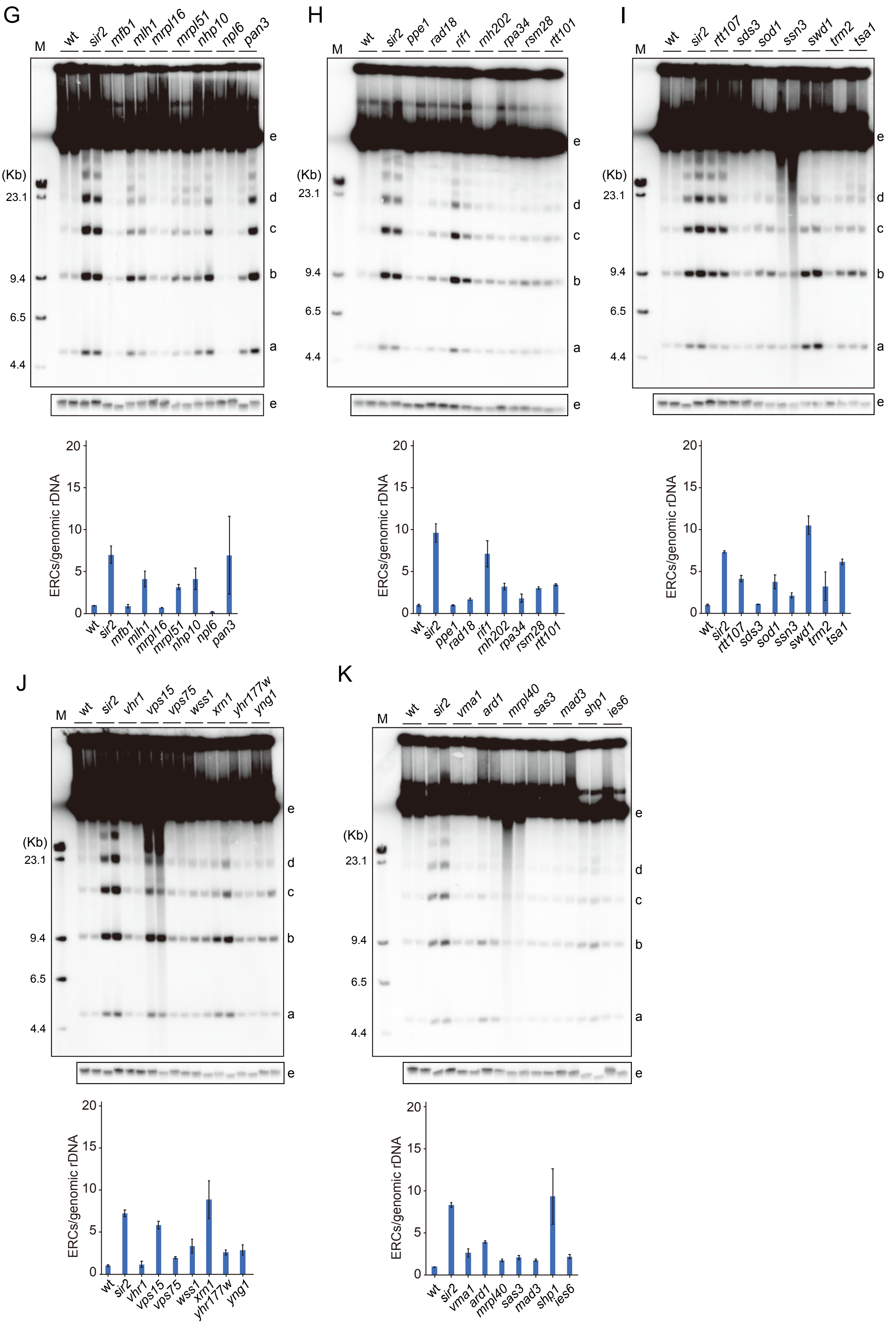

Hosoyamada_FigS2L

L

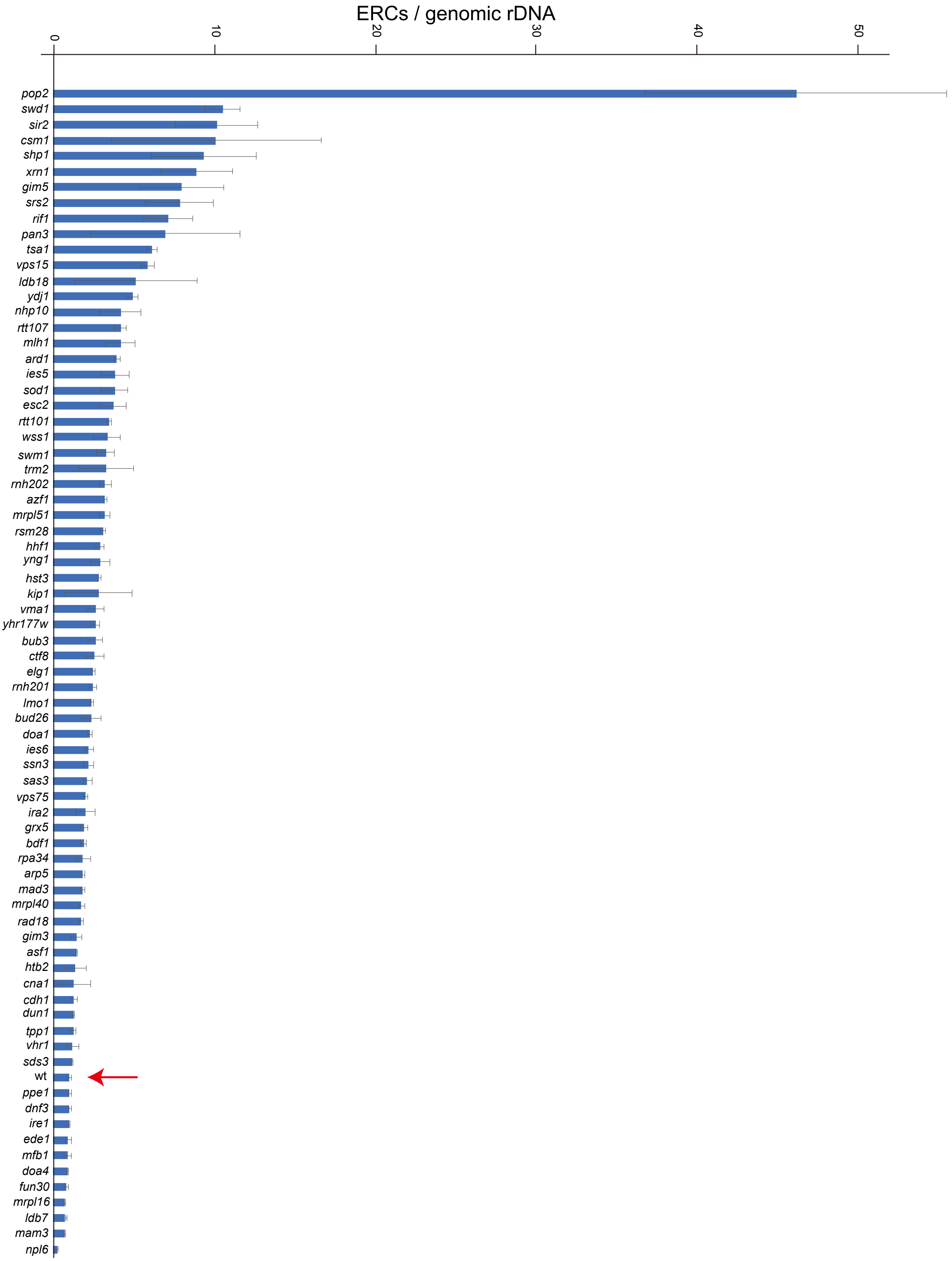

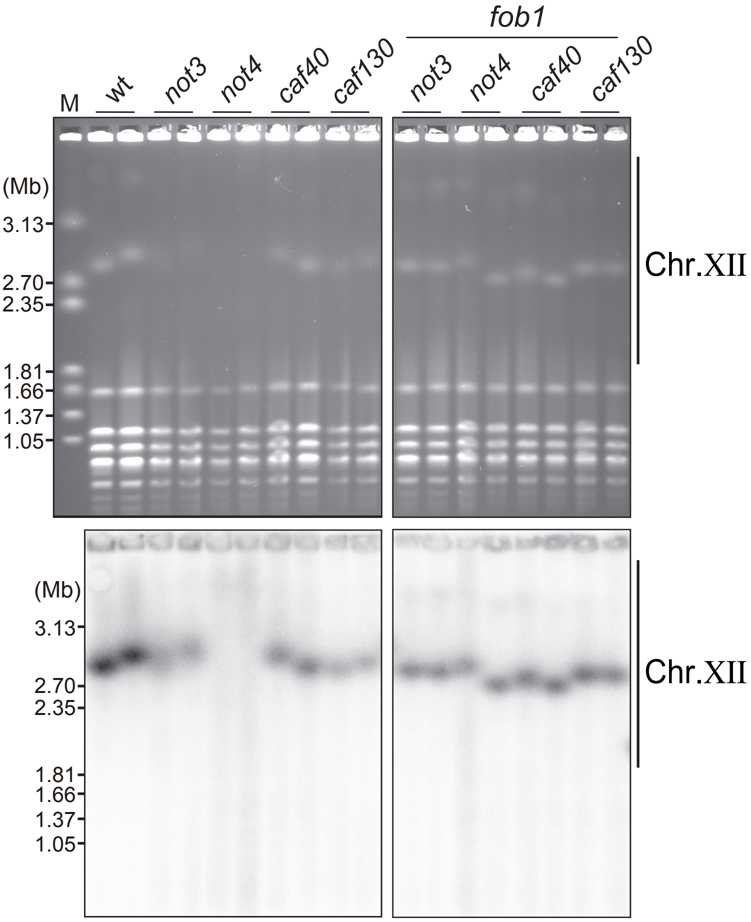
fob1
M
wt
not3
not4
caf40
caf130
not3
not4
caf40
caf130
(Mb)
3.13
2.70
2.35
1.81
1.66
1.37
1.05
Chr.XII
(Mb)
3.13
2.70
2.35
1.81
1.66
1.37
1.05
Chr.XII

Hosoyamada_FigS4

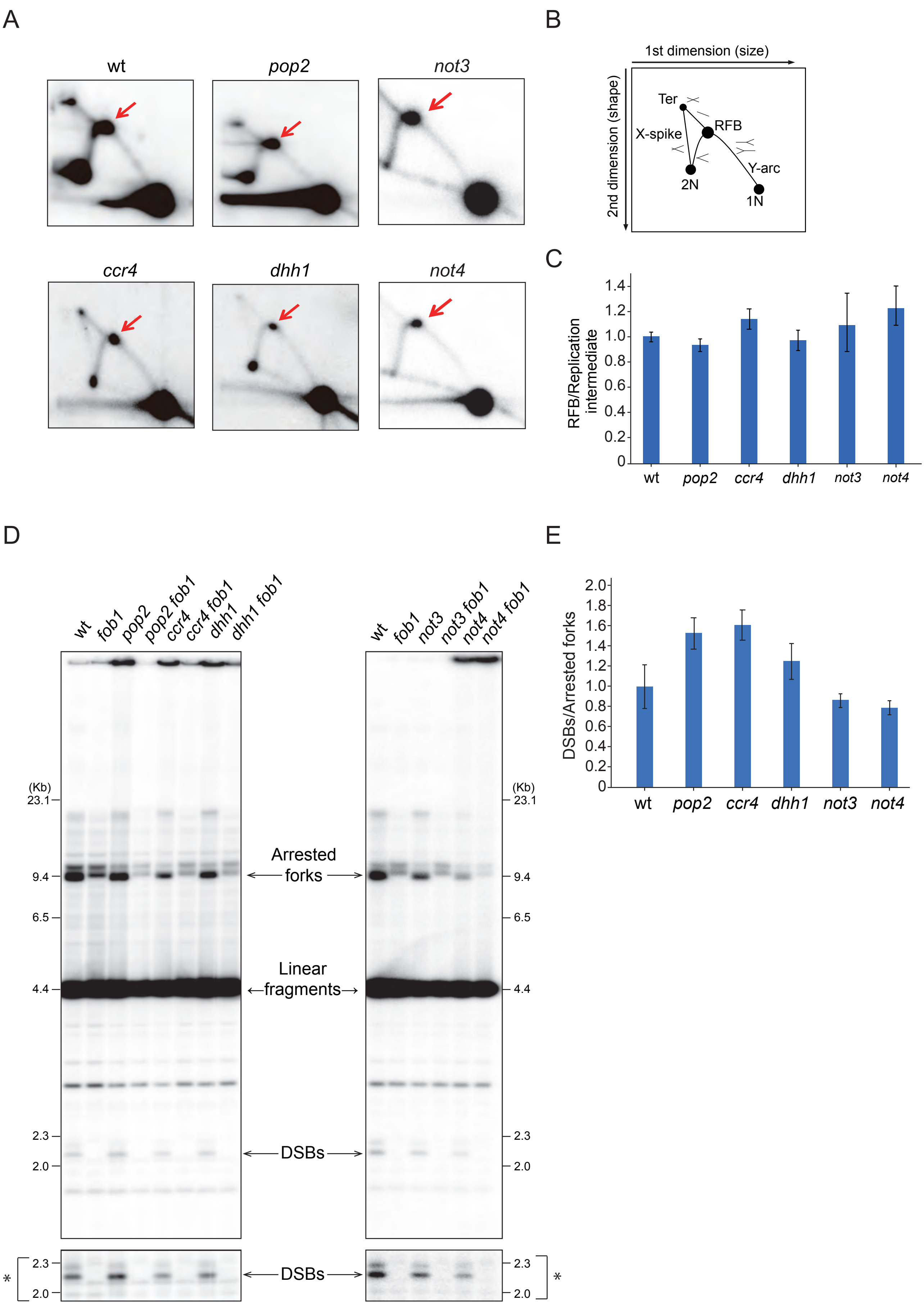

Hosoyamada_FigS5

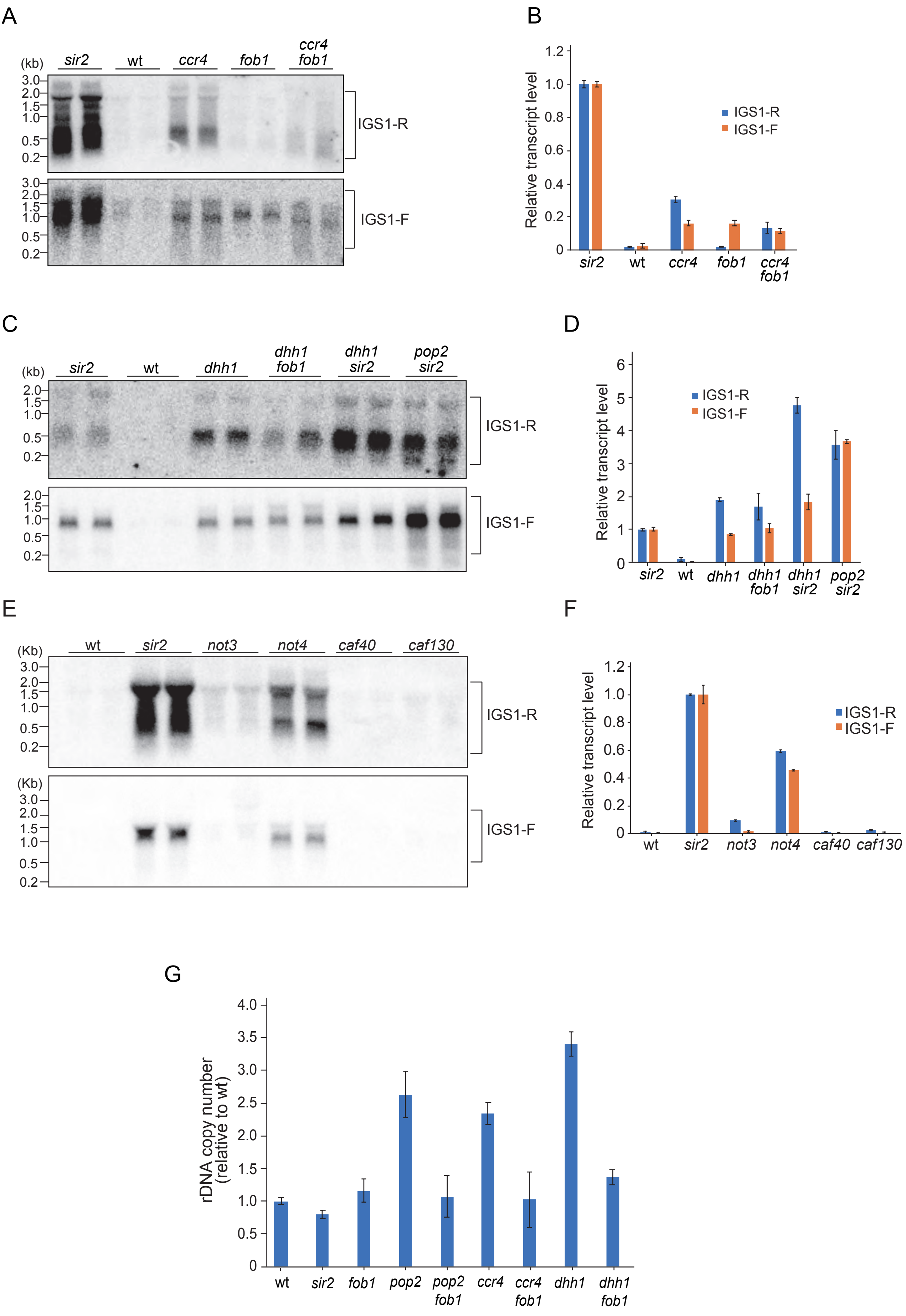

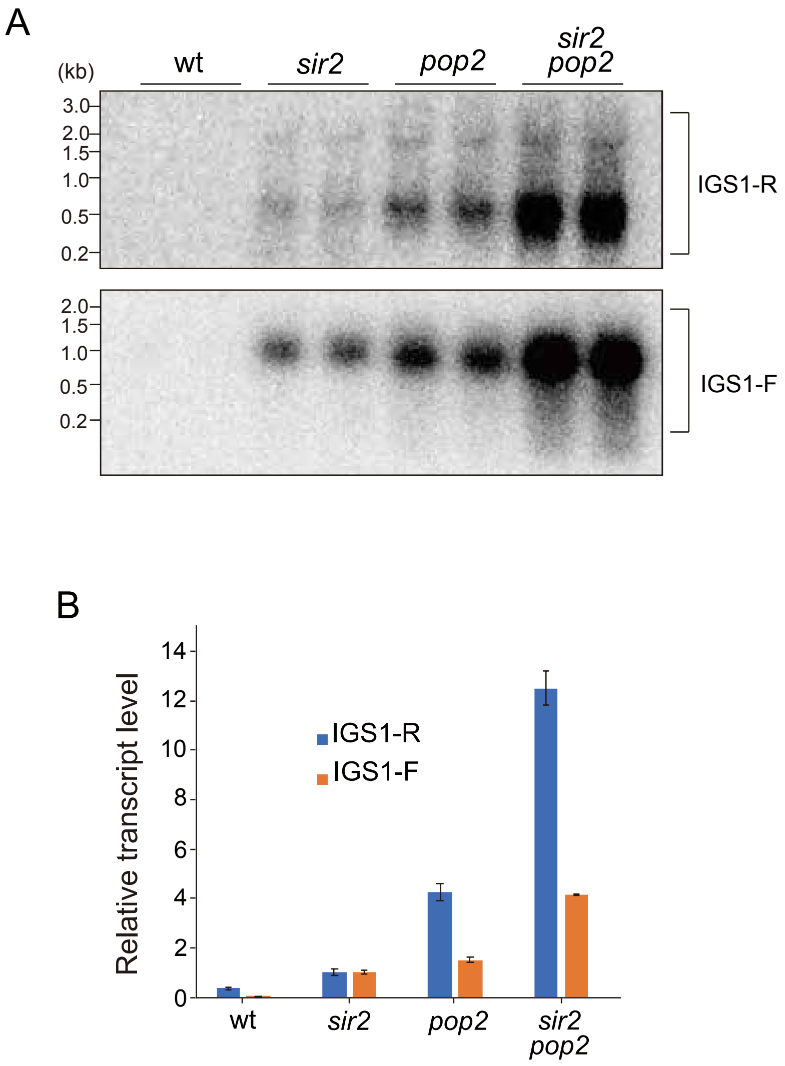
A
(kb)
wt
sir2
pop2
sir2
pop2
3.0
2.0
1.5
1.0
0.5
0.2
IGS1-R
2.0
1.5
1.0
0.5
0.2
IGS1-F
B
Relative transcript level
14
12
10
8
6
4
2
0
IGS1-R
IGS1-F
wt
sir2
pop2
sir2
pop2

Hosoiyamada_FigS7

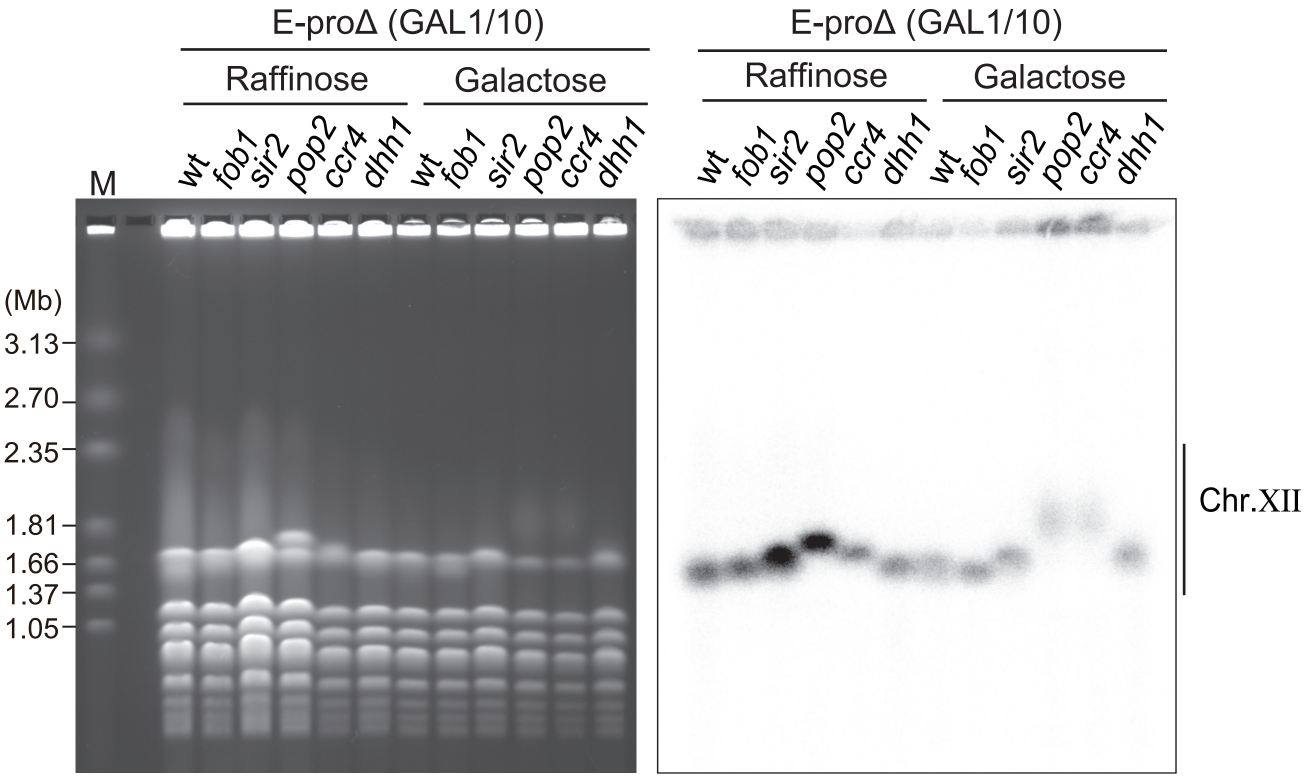

Hosoyamada_FigS8

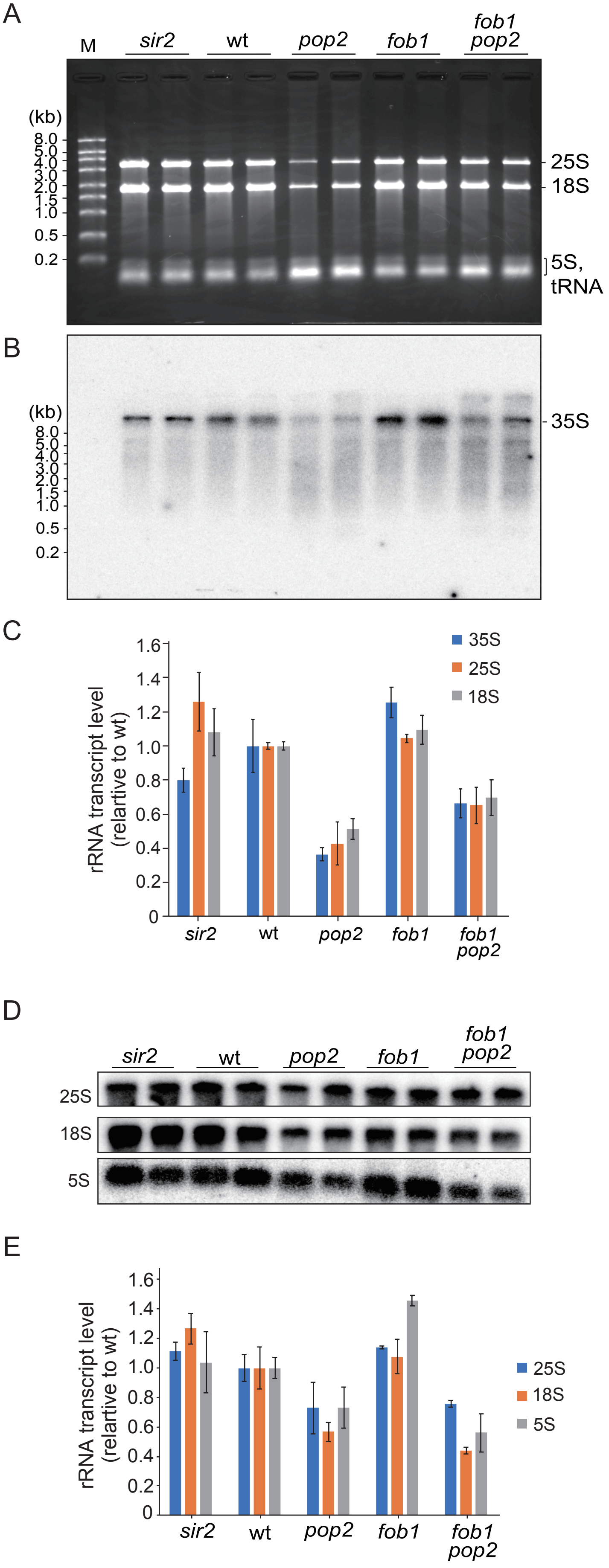

A

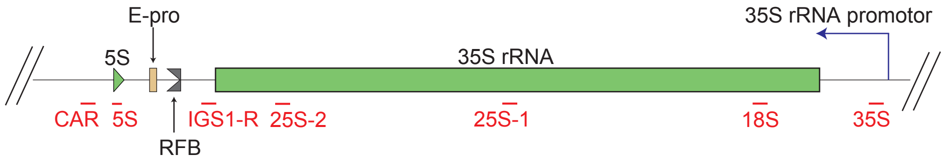
E-pro
35S rRNA promotor
5S
35S rRNA
CAR
5S
IGS1-R
25S-2
25S-1
18S
35S
RFB

B

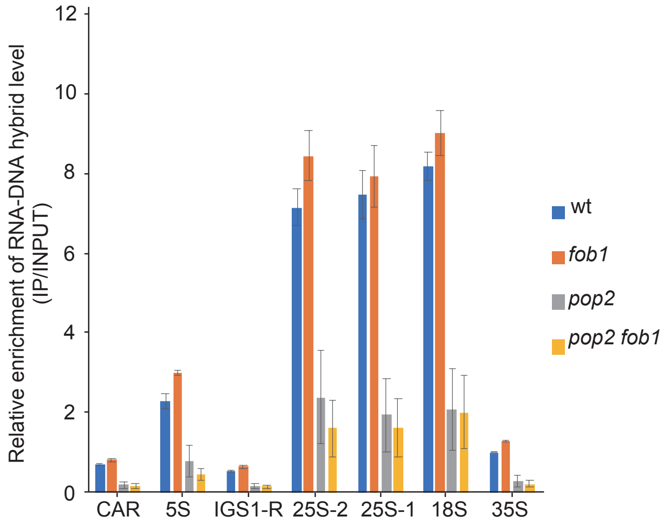
Relative enrichment of RNA-DNA hybrid level
(IP/INPUT)
12
10
8
6
4
2
0
CAR
5S
IGS1-R
25S-2
25S-1
18S
35S
wt
fob1
pop2
pop2 fob1

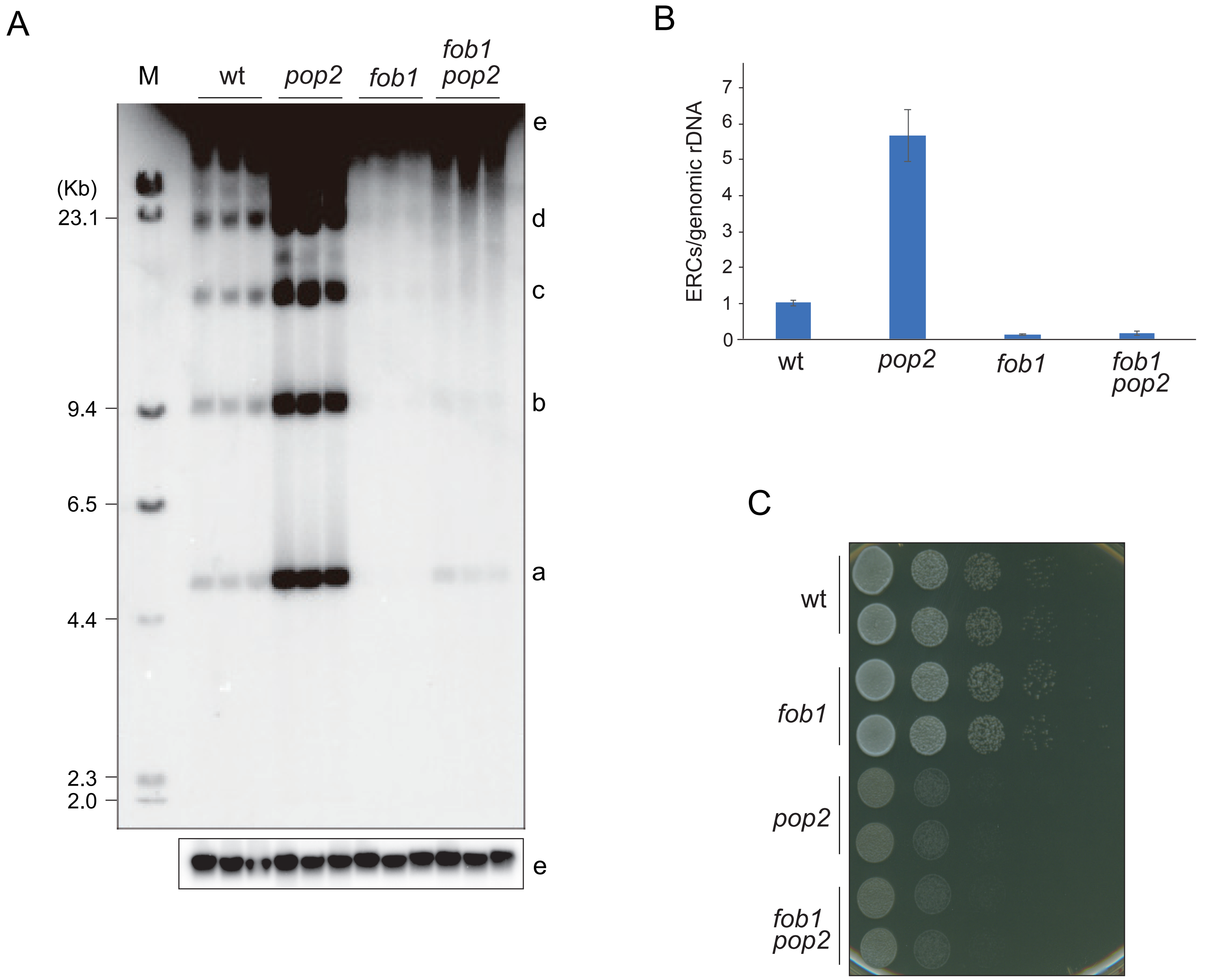
A
M
wt
pop2
fob1
fob1
pop2
(Kb)
23.1
9.4
6.5
4.4
2.3
2.0
e
d
c
b
a
e
B
ERCs/genomic rDNA
0
1
2
3
4
5
6
7
wt
pop2
fob1
fob1
pop2
C
wt
fob1
pop2
fob1
pop2

Supplementary Table S1. *S. cerevisiae* strains used in this study

Name	Genotype	Reference
NOY408-1b	*MATa*	Ref. S2
HSY30	*MATa, fob1::LEU2*	this study
HSY31	*MATa, sir2Δ::kanMX*	this study
TAK2004	*MATa, E-proΔ::GAL1/10-URA3*	Ref. S3
HSY190	TAK2004 with *pop2Δ::hphMX*	this study
HSY204	*MATa, pop2Δ::hphMX*	this study
HSY207	*MATα, fob1::LEU2, pop2Δ::hphMX*	this study
HSY210	*MATa, ccr4Δ::kanMX*	this study
HSY214	*MATa, fob1::LEU2, ccr4Δ::kanMX*	this study
HSY217	TAK2004 with *sir2Δ::hphMX*	this study
HSY244	TAK2004 with *fob1::LEU2*	this study
HSY262	*MATα, not3Δ::hphMX*	this study
HSY265	*MATα, fob1::LEU2, not3Δ::hphMX*	this study
HSY268	*MATα, not4Δ::kanMX*	this study
HSY271	*MATa, fob1::LEU2, not4Δ::kanMX*	this study
HSY274	*MATα, caf40Δ::kanMX*	this study
HSY277	*MATa, fob1::LEU2, caf40Δ::kanMX*	this study
HSY280	*MATa, caf130Δ::kanMX*	this study
HSY283	*MATα, fob1::LEU2, caf130Δ::kanMX*	this study
HSY292	*MATα, dhh1Δ::kanMX*	this study
HSY295	*MATα, fob1::LEU2, dhh1Δ::kanMX*	this study
HSY311	*MATa, fob1::LEU2, sir2Δ::hphMX*	this study
HSY315	*MATα, fob1::LEU2, pop2Δ::kanMX, sir2Δ::hphMX*	this study
HSY337	*MATα, sir2Δ::hphMX, pop2Δ::kanMX*	this study
HSY390	TAK2004 with *dhh1Δ::kanMX*	this study
HSY396	TAK2004 with *ccr4Δ::kanMX*	this study
HSY383	*MATa, MCD1-6HIS10FLAG-kanMX*	this study
HSY402	*MATα, MCD1-6HIS10FLAG-kanMX, sir2Δ::hphMX*	this study
HSY384	*MATa, MCD1-6HIS10FLAG-kanMX, pop2Δ::hphMX*	this study
HSY378	*MATa, SMC2-6HIS10FLAG-kanMX*	this study
HSY408	*MATa, SMC2-6HIS10FLAG-kanMX, sir2Δ::hphMX*	this study
HSY410	*MATa, SMC2-6HIS10FLAG-kanMX, pop2Δ::hphMX*	this study
HSY183	*MATa/α, fob1::LEU2/FOB1, pop2 Δ ::hphMX/POP2*	this study
HSY185	*MATa/α, fob1::LEU2/FOB1, ccr4 Δ ::kanMX/CCR4*	this study

All of the strains are *ade2-1, ura3-1, his3-11, 15, trp1-1, leu2-3, 112, can1-100*.

Supplementary Table S2. Oligonucleotides used in this study

Name	Sequence
HS405	ATGCCTGCAGGTCGACTCAAAACGGACAACCGGTGTATAATAT
HS406	GTACCCGGGGATCCTCTAAACAATAGTAACAGTGGACAGGAAA
HS411	GAGTCGACCTGCAGGCATGCAAGCT
HS412	TAGAGGATCCCCGGGTACCGAGCTC
HS409	CATGCCTGCAGGTCGACTTCTCACTGTTACAATGTATACTGCT
HS410	TACCCGGGGATCCTCTAGTTTGCAATCTGGTATGCTTTCTTGC
HS413	AGTCGACCTGCAGGCATGCAAGCTT
HS414	CTAGAGGATCCCCGGGTACCGAGCT
HS533	ATCGCCACTGCCTTTGTGGGGACTTTGGCT
HS534	AAAGGCAGTGGCGATTGACACATGATTATA
HS535	TTACAAGCCGTGGAGTCTAAGACTTTT
HS536	CTCCACGGCTTGTAAACACAACAGATC
HS204	CATTTCCTATAGTTAACAGGACATGCC
HS205	AATTCGCACTATCCAGCTGCACTC
HS206	ACGAACGACAAGCCTACTCG
HS207	AAAAGGTGCGGAAATGGCTG
HS210	GGCGAGGTTCAGAAAACTGTCG
HS211	AAACGGCAAGAATGCGTTGTTTG
HS393	GCAGCACCTGAGTTTCGCGTATGGT
HS394	TCCTCCACCCATAACACCTCTCACT
HS391	CGGGCGTGCTTGTGGACTGCTTGGT
HS392	GGCGCCGAAGCTCCCACTTATTCTA
HS387	TGCTCGAATATATTAGCATGGAATA
HS388	CGGCCATGCACCACCACCCACAAAA
HS274	AGGAATATCGGAGGAGAATATTGTT
HS275	TTCTAGTTTCTTGGCTTCCTATGCT
HS266	TGCAAAGATGGGTTGAAAGAGAA
HS296	CCGCGTCGCCGCGTCGCCAAAAAT
HS266	TGCAAAGATGGGTTGAAAGAGAA
HS275	TTCTAGTTTCTTGGCTTCCTATGCT
HS270	TGTCTTCCCATCTATCGTCGGTA
HS271	AGGGTTCATTGGAGCTTCAGTCA
HS542	ATGGTAACTTGAATGCAAAGAACGG
HS543	ATCAACAAGGTCTCTGACAACACAT
HS397	GTTGAAGACAAGTTCGAAAAGAGTT
HS398	GTCAATTAAACACGCTGTATAGAGA
HS431	CCACGATGAGACTGTTCAGG
HS432	GTCGCTAGGTGATCGTCAGA

HS435	TCCAATTGTTCCTCGTTAAG
HS436	ATTCAGGGAGGTAGTGACAA
HS445	TTACACCCAAACACTCGCAT
HS446	GACTGAGGACTGCGACGTAA
HS449	ACGCTTACCGAATTCTGCTT
HS450	CGTTCATAGCGACATTGCTT
HS453	ACTCATGTTTGCCGCTCTG
HS454	TGCAAAGATGGGTTGAAAGA
HS457	ACCTGCGTTTCCGTTAACT
HS458	AGTTGATCGGACGGGAAAC
HS459	GCCACCATCCATTTGTCTTT
HS460	TGAAAGTTGGTCGGTAGGTG
CUP1-fw	TGAAGGTCATGAGTGCCAAT
CUP1-rv	TTCGTTTCATTTCCCAGAGCA
